# Modeling Phage–Antibiotic Synergy, Innate Immunity, and Phage Resistance in Multidrug-Resistant *Acinetobacter baumannii*

**DOI:** 10.3390/antibiotics15090919

**Published:** 2026-09-17

**Authors:** Alma Karen Orozco-Ochoa, José Benigno Valdez-Torres, Jean Pierre González-Gómez, Nohelia Castro-del Campo, Cristóbal Chaidez-Quiroz

**Affiliations:** Laboratorio Nacional para la Investigación en Inocuidad Alimentaria (LANIIA), Centro de Investigación en Alimentación y Desarrollo, A.C. (CIAD), Carretera a Eldorado Km 5.5, Campo El Diez, Culiacán 80110, Mexico; aorozco222@estudiantes.ciad.mx (A.K.O.-O.); jvaldez@ciad.mx (J.B.V.-T.); jean.gonzalez@ciad.mx (J.P.G.-G.); ncastro@ciad.mx (N.C.-d.C.)

**Keywords:** host–pathogen interactions, antimicrobial resistance, phage susceptibility, phage–antibiotic synergy, mathematical model, computational modeling

## Abstract

**Background/Objectives:** Antimicrobial resistance has been recognized as a major global health threat, with multidrug-resistant *Acinetobacter baumannii* identified as one of the most critical pathogens. To address the limitations of conventional antibiotics, phage therapy has been proposed as a complementary or alternative intervention. In this study, experimental data were integrated into a deterministic differential-equation-based model to capture phage–bacteria–antibiotic–host immune system interactions. **Methods:** The model extended a previous phage–host immune system synergy framework by incorporating phage–antibiotic synergy and a time-dependent reduction in phage adsorption as a phenomenological representation of population-level reduction in phage susceptibility. This formulation does not explicitly model the molecular mechanisms or evolutionary emergence of resistance. In vitro observations of phage-induced resensitization to ceftazidime informed model parameterization, while remaining parameters were estimated from experimental observations or literature values. Simulations evaluated bacterial dynamics under phage-only, antibiotic-only, and immunity-only conditions, as well as combined therapeutic scenarios. **Results:** Model predictions indicated the greatest bacterial reduction when phages, antibiotics, and host innate immunity acted together. Phage–antibiotic synergy further enhanced predicted bacterial clearance, particularly for ceftazidime-resistant populations, while a population-level reduction in phage susceptibility was predicted approximately 4 h post-infection, consistent with experimental observations. Combined scenarios involving continuous antibiotic infusion, phage plus host immunity, or low-dose antibiotic regimens predicted accelerated bacterial declines when synergistic interactions were active. **Conclusions:** This framework integrates experimental observations with mathematical modeling to explore therapeutic interactions and temporal changes in phage susceptibility, while assessing parameter sensitivity and guiding future experimental and preclinical studies.

## 1. Introduction

Antimicrobial resistance (AMR) is one of the most serious global public health threats of this century [1]. Although concern about AMR emerged soon after the introduction of antibiotics, a global review to assess its magnitude and define strategies to combat rising drug resistance began only in 2014 [2]. AMR refers to the ability of microorganisms to withstand the effects of antimicrobial agents that would otherwise inhibit or eliminate them, thereby reducing the effectiveness of standard treatments. The emergence and dissemination of AMR are driven by multiple factors, including excessive and inappropriate antimicrobial use, inadequate infection prevention and control, poor sanitation, weak regulatory frameworks, and insufficient surveillance [3,4,5]. The continued spread of multidrug-resistant (MDR) pathogens further limits therapeutic options and increases the risks of treatment failure, prolonged illness, healthcare burden, and mortality [5]. AMR also represents a One Health challenge, as antimicrobial-resistant bacteria and resistance determinants can circulate among human, animal, food, and environmental compartments [5,6]. Recent estimates suggest that by 2050, approximately 2 million people could die annually from drug-resistant infections, primarily individuals aged 70 and over [7]. A major contributor to this burden is the rise of multidrug-resistant Gram-negative bacteria, with carbapenem-resistant *Enterobacteriaceae* and *Acinetobacter baumannii* posing the greatest threat to human health [8].

The increasing prevalence of multidrug-resistant organisms necessitates the development of innovative intervention strategies beyond conventional antibiotics. Phage therapy, using viruses that specifically target and kill bacteria, offers a promising alternative [9]. Phages are effective against both antibiotic-sensitive and -resistant bacteria [10,11] and have shown efficacy in treating a wide range of acute and chronic infections, including biofilm-associated infections and severe conditions such as sepsis, meningitis, and pneumonia [12,13,14,15,16,17]. However, phage-mediated lysis can alter bacterial populations and select phage-resistant mutants. Combination strategies with antibiotics have been explored to address this, though outcomes vary and underlying mechanisms remain incompletely understood [18,19,20,21].

Translating phage therapy into clinical products faces regulatory and experimental challenges [22]. Central to these challenges is understanding the complex tripartite interactions among phages, bacteria, and eukaryotic hosts, as stochastic evolution and co-evolution make outcomes difficult to predict [23]. In vivo conditions differ from in vitro settings due to factors such as the immune system, anatomical barriers, host health, and immune status [23,24,25]. The host immune system can influence phage efficacy [26,27], while the emergence of phage-resistant bacteria under selection pressure can alter fitness, virulence, and, in some cases, confer cross-resistance to other phages or antibiotics [28,29,30]. Phage resistance may arise through alterations or loss of bacterial surface structures involved in phage adsorption, including receptors associated with the bacterial envelope. Such changes may also impose fitness costs or, depending on the affected structures, alter susceptibility to antibiotics. Phages can also adapt to overcome bacterial defenses [31].

Deterministic mathematical models combined with experimental data offer a complementary approach to understanding these interactions. While models are simplified abstractions and cannot fully capture biological complexity [23,32], they can provide mechanistic insights and generate testable hypotheses to inform subsequent experimental and preclinical studies. Early models described viral particle changes within host cells, aiding the interpretation of one-step growth experiments, including latency periods and burst size [33]. More recent models focus on specific interactions among phages, bacteria, and the immune system. One model describes immunophage synergy, whereby phages and the host immune system act together to eliminate bacteria, accounting for phage lysis, density-dependent immune evasion, and immune capacity, thereby guiding the optimization of phage therapy strategies [26]. Extending this, another model analyzed combined phage–antibiotic therapy in *Pseudomonas aeruginosa*, demonstrating that the combination outperforms either treatment alone, with immune interactions enhancing efficacy [18].

In this study, in vitro data were integrated within a deterministic system of differential equations to capture the key dynamics of phage–bacteria–host immune system and guide phage–antibiotic combination therapies. The model builds on the phage–host immune system synergy model developed by [26] and incorporates phage–antibiotic interactions to simulate their potential contribution to bacterial clearance, particularly by resensitizing antibiotic-resistant bacteria, while considering immune cell activity. Phages target multidrug-resistant *Acinetobacter baumannii*, enabling antibiotics and immune effector cells (represented by neutrophils [26,34]) to act on bacterial populations, affecting both susceptible and resistant bacteria. The model also considers a time-dependent reduction in phage adsorption to represent the population-level effect of reduced phage susceptibility over time and its impact on infection dynamics. Consistent with previous experimental observations, *A. baumannii* strain AbAK03 exhibits resistance to ceftazidime, whereas phage exposure promotes resensitization to antibiotic activity and is associated with reduced phage susceptibility approximately 4 h post-infection [20]. These experimental observations were represented in the model through the phage–antibiotic synergy term and a time-dependent reduction in phage adsorption, respectively, rather than through an explicit genetic or population-based model of resistance evolution. This study aims to use mathematical modeling to investigate the predicted effects of combination therapy on multidrug-resistant bacterial dynamics under active immune responses, with a focus on phage–antibiotic synergy, innate immunity, and temporal changes in phage susceptibility.

**Figure 1:**
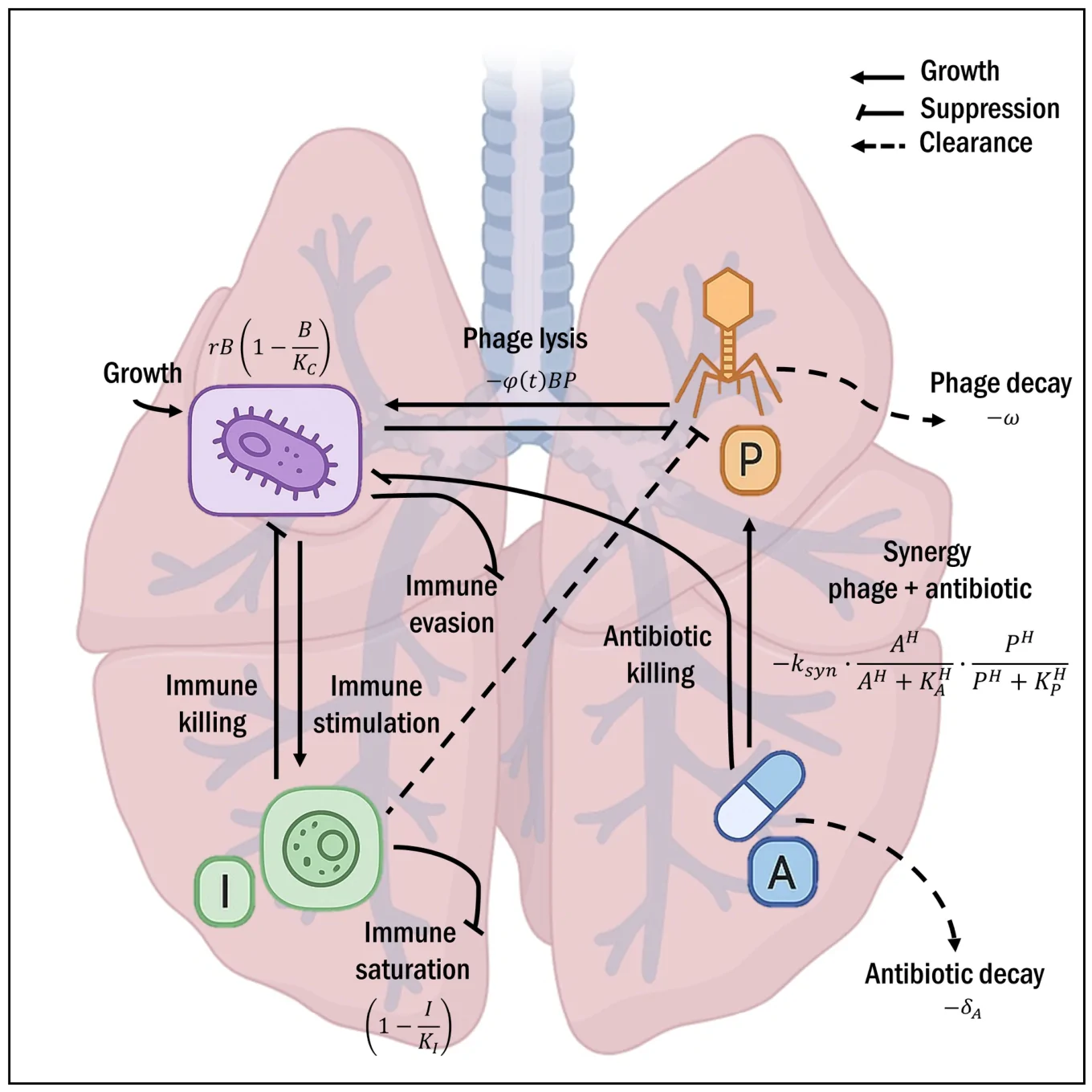
Schematic diagram of a phage–antibiotic combination model incorporating immune saturation, bacterial immune evasion, and a time-dependent reduction in phage susceptibility. Created by the authors.

**Phage–Antibiotic Combination Model**. This phage therapy model integrates the interactions among bacteria (B), phage (P), antibiotic (A), and the innate immune response (I) (Figure 1). The model represents a simplified scenario of acute infection involving *Acinetobacter baumannii* strain AbAK03 [35], assuming that bacterial clearance is primarily driven by the host innate immune system. In this model, the bacterial population replicates and can be infected and lysed by phage Indie [20], while free phage particles decay when outside host cells. The presence of bacteria activates the innate immune response, which in turn eliminates bacterial cells, as supported by a previous theoretical study [26]. Phages further contribute to bacterial killing in the model, and the experimentally observed phage-associated resensitization to antibiotic activity was incorporated as a synergistic interaction, allowing the model to explore its potential contribution to antimicrobial efficacy. This representation captures the observed functional interaction between phage exposure and antibiotic activity but does not imply a direct mechanistic link between phage resistance and antibiotic resensitization. This approach builds upon fundamental principles established in previous experimental studies [20,21]. In addition, it incorporates two biological processes not explicitly represented in previous models, phage–antibiotic synergy and a time-dependent reduction in phage adsorption representing a population-level reduction in phage susceptibility, both parameterized using in vitro experimental data obtained from phage–antibiotic combination assays reported by [20].

The dynamics of the interactions illustrated in Figure 1 are modeled by a system of differential equations (Equations (1)–(4)), which captures the time-dependent behavior of bacteria, phage, antibiotic, and the host immune system. The system integrates bacterial growth, phage infection, immune response, antibiotic dynamics, and phage–antibiotic synergy.

**Equation (1). Immune system model**

(1) $$\frac{dI}{dt}=\alpha \left(\frac{B}{B+K_{N}}\right)\left(1-\frac{I}{K_{I}}\right)I$$

where

*I* = innate immune cell population (neutrophils) present at time *t* ($cell\;{mL}^{-1}$).

B = bacterial population density.

α = maximum immune cell proliferation rate.

$K_{I}$ = carrying capacity of the immune response.

$K_{N}$ = bacterial density at which immune activation reaches half of its maximum value.

This equation describes the dynamics of the host innate immune system. The immune cell population (*I*) expands at a maximum rate α but is constrained by the carrying capacity $K_{I},$ represented by the logistic term $\left(1-\frac{I}{K_{I}}\right)$. Immune simulation depends on bacterial burden (B) through the saturating function $\frac{B}{B+K_{N}}$, reflecting the increasing activation of immune cells as bacterial density rises. The parameter $K_{N}$ corresponds to the bacterial concentration at which the immune response reaches 50% of its maximum simulation level. This formulation captures both the finite growth potential of the immune response and its dependence on bacteria presence [26,36].

**Equation (2). Antibiotic model**

(2) $$\frac{dA}{dt}=-\delta_{A}A$$

where

*A* = antibiotic concentration at time *t* ($\mu g\;{mL}^{-1}$).

$\delta_{A}$ = antibiotic elimination or degradation rate constant ($h^{-1}$).

$A_{0}$ = initial antibiotic concentration.

Equation (2) represents the dynamic antibiotic model used when antibiotic elimination or degradation is considered. Under this formulation, the antibiotic concentration (A) decreases exponentially over time at a rate determined by the antibiotic elimination or degradation rate constant $\delta_{A}$. The term $-\delta_{A}A$ represents the loss of antibiotics due to degradation, clearance, or other elimination processes. In simulations in which antibiotic exposure was intended to represent a constant concentration, the antibiotic concentration was fixed at the initial concentration ($A=A_{0}$) throughout the simulation, and Equation (2) was not dynamically solved. Thus, these simulations represent constant antibiotic exposure rather than antibiotic elimination or degradation.

**Equation (3). Phage model**

(3) $$\frac{dP}{dt}=\beta \varphi (t)BP-\omega P$$

where

P = phage concentration at time *t*.

β = burst size.

φ(t) = phage adsorption rate at time *t*.

B = bacterial population density.

ω = natural phage decay rate constant.

This equation describes the dynamics of the phage population. The phage concentration (P) increases through successful infection and lysis of susceptible bacteria at a rate determined by φ(t)BP. Each infection event produces new phage particles according to the burst size β, represented by the term βφ(t)BP. Simultaneously, phage particles are removed from the system at a rate ωP, which accounts phage decay and removal within the host [27]. The parameter ω was estimated from previously reported experimental in vivo phage decay data [27]. This formulation captures the balance between phage replication and phage removal within the host.

The phage adsorption rate, φ(t), is modeled as a sigmoidal function that gradually decreases from an initial value $\varphi_{0}$ to a lower value $\varphi_{1}$ over time:

(4) $$\varphi (t)=\varphi_{1}+\frac{\varphi_{0}-\varphi_{1}}{1+e^{k(t-t_{c})}}$$

where $\varphi_{0}$ and $\varphi_{1}$ represent the initial and final adsorption rates, respectively, $t_{c}$ denotes the characteristic time at which the reduction in adsorption is centered, and k controls the steepness of the transition. This sigmoidal function provides a phenomenological representation of the population-level reduction in phage susceptibility by describing the progressive reduction in phage adsorption efficiency over time. It is not intended to represent the mechanisms or evolutionary dynamics underlying the emergence of phage resistance; rather, it captures their net effect on phage–bacteria interactions. Initially, the adsorption rate remains close to $\varphi_{0}$, whereas as bacterial susceptibility to phage infection decreases, it progressively approaches $\varphi_{1}$. Thus, the decrease in $\varphi \left(t\right)$ serves as a mathematical representation of the loss of bacterial susceptibility to phage infection. Biologically, reduced phage adsorption may result from modification or loss of bacterial surface structures that function as phage receptors [37], including alterations in components of the bacterial envelope. The present model does not explicitly incorporate a fitness cost associated with phage resistance. Resistant bacteria are therefore assumed to retain the same intrinsic growth parameters as the susceptible bacterial population. This simplification was adopted because quantitative measurements of growth rate or competitive fitness for phage-resistant derivatives were not available for model parameterization. In biological systems, phage resistance may impose fitness costs through alterations in surface structures, membrane composition, or other cellular functions, whereas compensatory changes may reduce these costs over time [37]. Consequently, the present formulation should be interpreted as evaluating the consequences of reduced phage susceptibility under a neutral fitness-cost assumption rather than predicting the evolutionary fitness of resistant variants. Because these mechanisms and their fitness consequences were not experimentally resolved in the present study, they were not explicitly represented in the model.

**Equation (4). Integrated phage, antibiotic and immune system model**

(5) $$\frac{dB}{dt}=rB\left(1-\frac{B}{K_{C}}\right)-\varphi \left(t\right)BP-\left(\frac{\epsilon IB}{1+\frac{B}{K_{D}}}\right)-k_{syn}\cdot \frac{A^{H}}{A^{H}+K_{A}^{H}}\cdot \frac{P^{H}}{P^{H}+K_{P}^{H}}\;$$

where

r = maximum bacterial growth rate.

$K_{C\;}$ = bacterial carrying capacity.

ϵ = immune clearance efficiency.

$K_{D}$ = bacterial density at which immune killing efficiency is reduced by 50%.

$k_{syn}$ = maximum bacterial clearance rate attributable to phage–antibiotic synergy (CFU ${mL}^{-1}$ $h^{-1}$).

$K_{A}\;$= antibiotic concentration required to achieve half of the maximal synergy effect ($\mu g\;{mL}^{-1}$).

$K_{P}\;$= phage concentration required to achieve half of the maximal synergy effect ($PFU\;{mL}^{-1}$).

H = Hill coefficient describing the degree of cooperativity.

This equation describes bacterial population dynamics under the combined effects of bacterial growth, phage infection, immune-mediated killing, and phage–antibiotic synergy. Bacterial growth follows logistic dynamics with a maximum growth rate r and carrying capacity $K_{C}$, represented by the term $rB\left(1-\frac{B}{K_{C}}\right)$. Bacterial reduction occurs through phage-mediated infection at a rate φ(t)BP, where φ(t) is the time-dependent phage adsorption rate. Additional bacterial clearance is mediated by the host innate immune response through the term $\frac{\epsilon IB}{1+\frac{B}{K_{D}}}$, where ϵ represents immune killing efficiency and $K_{D}$ accounts for the reduced effectiveness of immune cells at high bacterial densities.

The final term,

(6) $$k_{syn}\left(\frac{A^{H}}{A^{H}+K_{A}^{H}}\right)\left(\frac{P^{H}}{P^{H}+K_{P}^{H}}\right),$$

represents the additional bacterial clearance attributable to the synergistic interaction between antibiotics and phages. Here, $k_{syn}$ represents the maximum bacterial clearance rate attributable to the phage–antibiotic synergistic effect and has units of CFU ${mL}^{-1}$ $h^{-1}$, consistent with the units of $\frac{dB}{dt}$. The two saturation terms, $\frac{A^{H}}{A^{H}+K_{A}^{H}}$ and $\frac{P^{H}}{P^{H}+K_{P}^{H}}$, are dimensionless because A and $K_{A}$ have the same units, as do P and $K_{P}$. Therefore, the complete synergy term has units of CFU ${mL}^{-1}$ $h^{-1}$, ensuring dimensional consistency with the bacterial population equation. The parameters $K_{A}$ and $K_{P}$ correspond to the antibiotic and phage concentrations required to achieve half of the maximal synergy effect, respectively. The Hill coefficient H determines the steepness of the response, with H=1 indicating a hyperbolic relationship and higher values representing positive cooperativity. This formulation captures the nonlinear and saturable enhancement of bacterial clearance resulting from the combined action of antibiotics and phages [18,38]. Importantly, the synergy term represents the experimentally observed enhancement of antibiotic-associated bacterial clearance following phage exposure and is not formulated as a consequence of the emergence or evolution of phage-resistant mutants. Therefore, the model evaluates these processes as interacting population-level effects rather than as a mechanistically coupled evolutionary pathway.

**Table 1:** Model parameters and their parameterization sources.

Symbol	Parameter	Value	Parameterization Source
r	Growth rate of bacteria at low density	1 h^−1^	This study; see [20]
$K_{C\;}$	Carrying capacity of bacteria	10^8^ CFU mL^−1^	This study; see [20]
$B_{0}$	Initial bacteria density	10^6^ CFU mL^−1^	This study; see [20]
β	Burst size of phage	13 PFU CFU^−1^	This study; see [20]
$\varphi_{0}$	Phage adsorption rate to bacteria at t = 0	6 × 10^−8^ mL·PFU^−1^·h^−1^	This study; see [20]
$\varphi_{1}$	Phage adsorption rate to bacteria over time	8 × 10^−10^ mL·PFU^−1^·h^−1^	This study; see [20]
ω	Phage decay/removal rate	0.069 h^−1^	Experimental in vivo phage decay data [27]
$P_{0}$	Initial phage dose	10^7^ PFU mL^−1^	This study; see [20]
α	Maximum growth rate of innate immune response	0.97 h^−1^	Fitting experimental neutrophil recruitment data [26,39]
$I_{0}$	Initial innate immune intensity	2.7 × 10^6^ cell mL^−1^	Fitting neutrophil recruitment data in models of acute lung injury [26,39]
$K_{I}$	Maximum capacity of innate immune response	2.4 × 10^7^ cell mL^−1^	Fitting neutrophil recruitment data in models of acute lung injury [26,39]
ϵ	Killing rate parameter of innate immune response	8.2 × 10^−8^ mL/h cell	Set ϵK_I_ so that it equals 1.97 h^−1^, the fitted maximum neutrophil killing rate in a murine pneumonia model [26,40]
$K_{D}$	Bacterial concentration at which innate immune response is half saturated	2.2 × 10^6^ CFU mL^−1^	Estimated from reference [40]
$K_{N}$	Bacterial concentration when innate immune response growth rate is half its maximum	1 × 10^5^ CFU mL^−1^	Estimated from reference [26]
$A_{0}$	Initial dose of antibiotic	10 µg mL^−1^	Estimated from reference [21]
$\delta_{A}$	Antibiotic elimination/degradation rate	0.05 h^−1^	This study; see [20]
$k_{syn}$	Maximum bacterial clearance rate due to phage–antibiotic synergy	42.5 CFU·mL^−1^·h^−1^	This study; see [20]
$K_{A}$	Antibiotic synergy constant	5.81 µg mL^−1^	This study; see [20]
$K_{P}$	Phage synergy constant	1 × 10^6^ PFU mL^−1^	This study; see [20]
$t_{c}$	Characteristic time of the reduction in phage adsorption	4 h	This study; see [20]. Estimated from the experimentally observed reduction in phage susceptibility
H	Hill coefficient	1	Estimated from reference [41].
k	Sigmoid adsorption transition slope	3 h^−1^	This study; see [20]

## 2. Results

### 2.1. Bacterial Dynamics Under Individual Treatments

**Figure 2:**
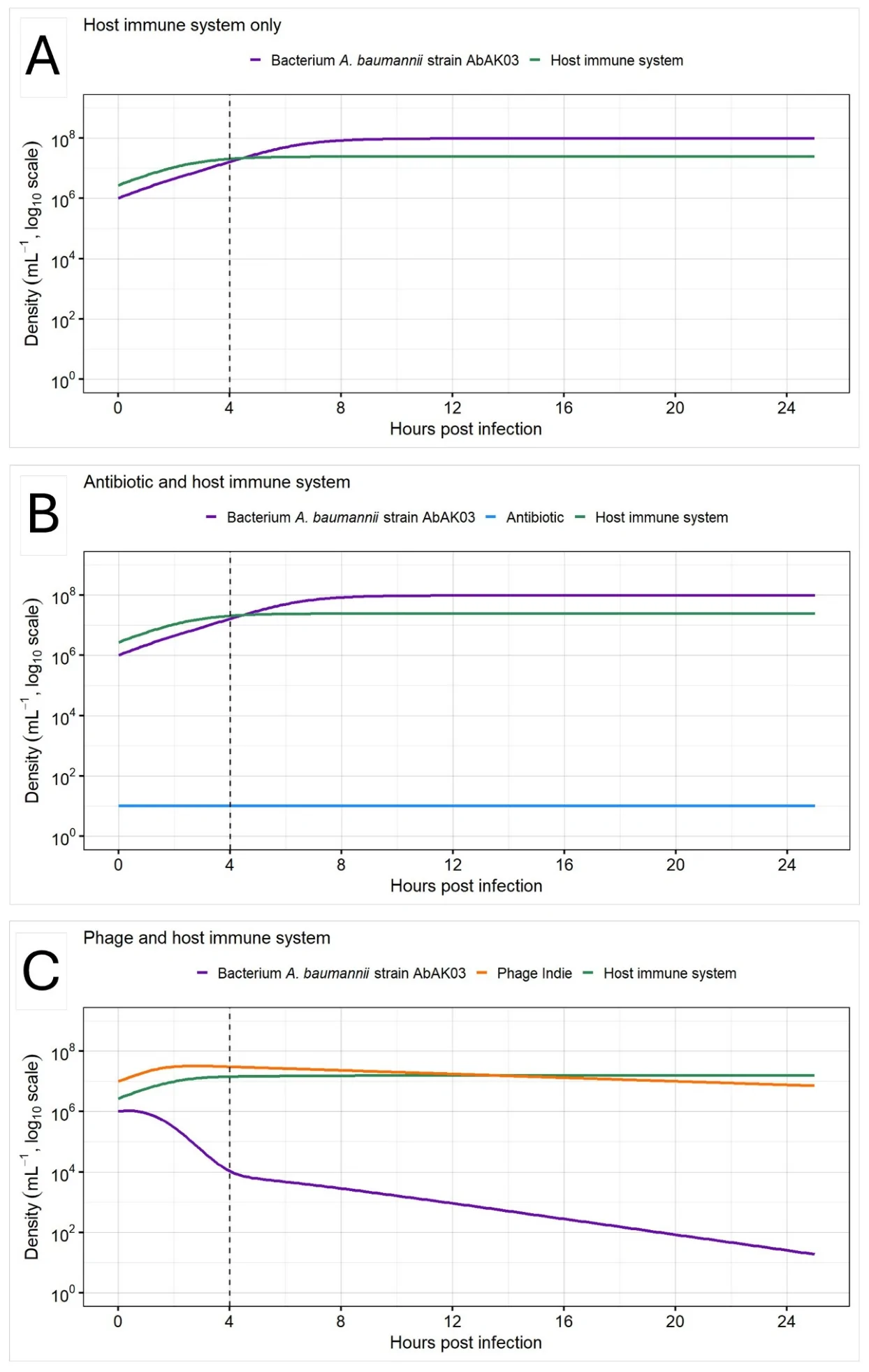
Model 1: Dynamics of bacterial infection under different treatment combinations. Simulation of bacteria, phage, host immune system, and antibiotic dynamics over 25 h for three scenarios: (**A**) host immune system-only, (**B**) antibiotic and host immune system, and (**C**) phage and host immune system. Initial conditions were $B_{0}$ = 10^6^ CFU mL^−1^, $P_{0}$ = 10^7^ PFU mL^−1^, $I_{0}$ = 2.7 × 10^6^ cells mL^−1^, and $A_{0}$ = 10 µg mL^−1^ (ceftazidime). For scenario (**B**), the antibiotic concentration was fixed at A = $A_{0}$ = 10 µg mL^−1^ throughout the simulation, representing constant antibiotic exposure. The dashed vertical line at 4 h indicates the transition time ($t_{c}$) associated with the experimentally observed reduction in phage susceptibility and the corresponding modeled decline in phage adsorption efficiency. The *y*-axis is plotted on a logarithmic scale to facilitate visualization of temporal changes in all model variables. The model predicts that phage-mediated therapy (**C**) results in a marked reduction in bacterial density compared to the antibiotic and host immune system scenario (**B**), which shows limited bacterial control due to the intrinsic resistance of *A. baumannii* strain AbAK03 to ceftazidime. Colors indicate bacterial population (purple), phage (orange), host immune cells (green), and antibiotic concentration (blue).

In the first scenario (Figure 2A), representing infection dynamics under host immune system alone, phage and antibiotic inputs were set to zero ($P_{0}$ = 0 and $A_{0}$ = 0). This configuration isolates host-mediated bacterial control driven exclusively by innate immune effector cells (I), allowing evaluation of the intrinsic capacity of the host immune system to control bacterial proliferation in the absence of pharmacological or viral intervention. Bacterial density increased from 10^6^ to approximately 10^8^ CFU mL^−1^ over 25 h, while host immune cells (neutrophils) increased and stabilized at approximately 10^7^ cells mL^−1^, indicating insufficient immune control of bacterial growth.

In the second scenario (Figure 2B), corresponding to antibiotic treatment combined with the host immune system, phages were excluded ($P_{0}$ = 0), while antibiotic exposure was fixed at a constant concentration of $A=A_{0}=10$ µg mL^−1^ throughout the 25 h simulation. Under this simulation condition, antibiotic concentration was not allowed to decrease according to Equation (2); instead, it was held constant to represent continuous antibiotic exposure. This condition represents simulated ceftazidime exposure against the multidrug-resistant *A. baumannii* strain AbAK03, which was previously shown to be resistant to this antibiotic [20]. Bacterial density similarly increased to approximately 10^8^ CFU mL^−1^ over 25 h, indicating that ceftazidime alone was unable to control infection due to the intrinsic resistance phenotype of *A. baumannii* strain AbAK03, despite the presence of an active innate immune response.

In the third scenario (Figure 2C), phage therapy was simulated using phage Indie by maintaining an initial phage load at $P_{0}$ = 10^7^ PFU mL^−1^ while setting antibiotic concentration to zero ($A_{0}$ = 0). In this condition, the phage population is predicted to replicate within the bacterial population, as well as driving density-dependent infection and lysis dynamics. Bacterial density decreased from 10^6^ CFU mL^−1^ to approximately 10^1^ CFU mL^−1^ after 25 h, while phage density remained above 10^6^ PFU mL^−1^ throughout the simulation. The simulations predicted that phage therapy combined with the host immune system produced markedly enhanced bacterial clearance compared with the host immune system alone or antibiotic exposure in the absence of phages.

### 2.2. Dynamic Model of Bacterial Infection with Phage–Antibiotic Synergy and Host Immune System

Model 2 extends Model 1 by incorporating phage–antibiotic synergy together with the host immune system synergy described by [26]. The same ordinary differential equation (ODE) structure is retained, including bacterial growth, phage infection, antibiotic dynamics, and the host immune system, while additional nonlinear interaction terms are introduced to capture cooperative bacterial killing.

Bacterial growth follows a logistic formulation constrained by a carrying capacity $K_{C}=10^{8}$ CFU mL^−1^, and is reduced through phage predation, antibiotic activity, immune-mediated killing, and a phage–antibiotic synergy term modeled using Hill-type functions.

Phage adsorption dynamics were modeled as a time-dependent sigmoidal function (k=3 h^−1^), representing the experimentally observed population-level reduction in phage susceptibility through a population-level decline in adsorption efficiency centered at $t_{c}=4\;h$. This formulation is phenomenological and does not explicitly model the genetic or evolutionary emergence of phage resistance. The host immune system is activated in a bacterial-dependent manner with saturation effects, where $K_{D}=2.2\times 10^{6}$ CFU mL^−1^ represents half-maximal immune killing and $K_{N}=1\times 10^{5}$ CFU mL^−1^ defines the bacterial density associated with half-maximal immune activation [26]. Phages replicate through bacterial infection and decay at a natural rate ω=0.069 h^−1^. The synergistic interaction between phage and antibiotic is described by a maximum magnitude $k_{\mathrm{syn}}=42.5$ CFU·mL^−1^·h^−1^, with saturation constants $K_{A}=5.81$ µg mL^−1^ and $K_{P}=1\times 10^{6}$ PFU mL^−1^ [20,21,42,43]. These parameters were estimated from experimental phage–antibiotic combination data [20] rather than from an independent mechanistic measurement of synergy.

Simulations were performed over 25 h under two conditions: with and without phage–antibiotic synergy, using identical initial states (B_0_ = 10^6^ CFU mL^−1^, P_0_ = 10^5^ PFU mL^−1^, I_0_ = 2.7 × 10^6^ cells mL^−1^, A_0_ = 1.6 µg mL^−1^). For these simulations, the antibiotic concentration was fixed at A = A_0_ = 1.6 µg mL^−1^ throughout the 25 h simulation. Thus, Equation 2 was not dynamically solved in this analysis, and dA/dt was set to zero to represent sustained, constant subinhibitory antibiotic exposure. In the non-synergistic scenario, $k_{\mathrm{syn}}=0$, effectively removing cooperative clearance effects while preserving standard phage predation and immune-mediated killing. In contrast, the synergistic condition was activated with $k_{\mathrm{syn}}=42.5$, enabling enhanced bacterial clearance through combined phage–antibiotic activity.

**Figure 3:**
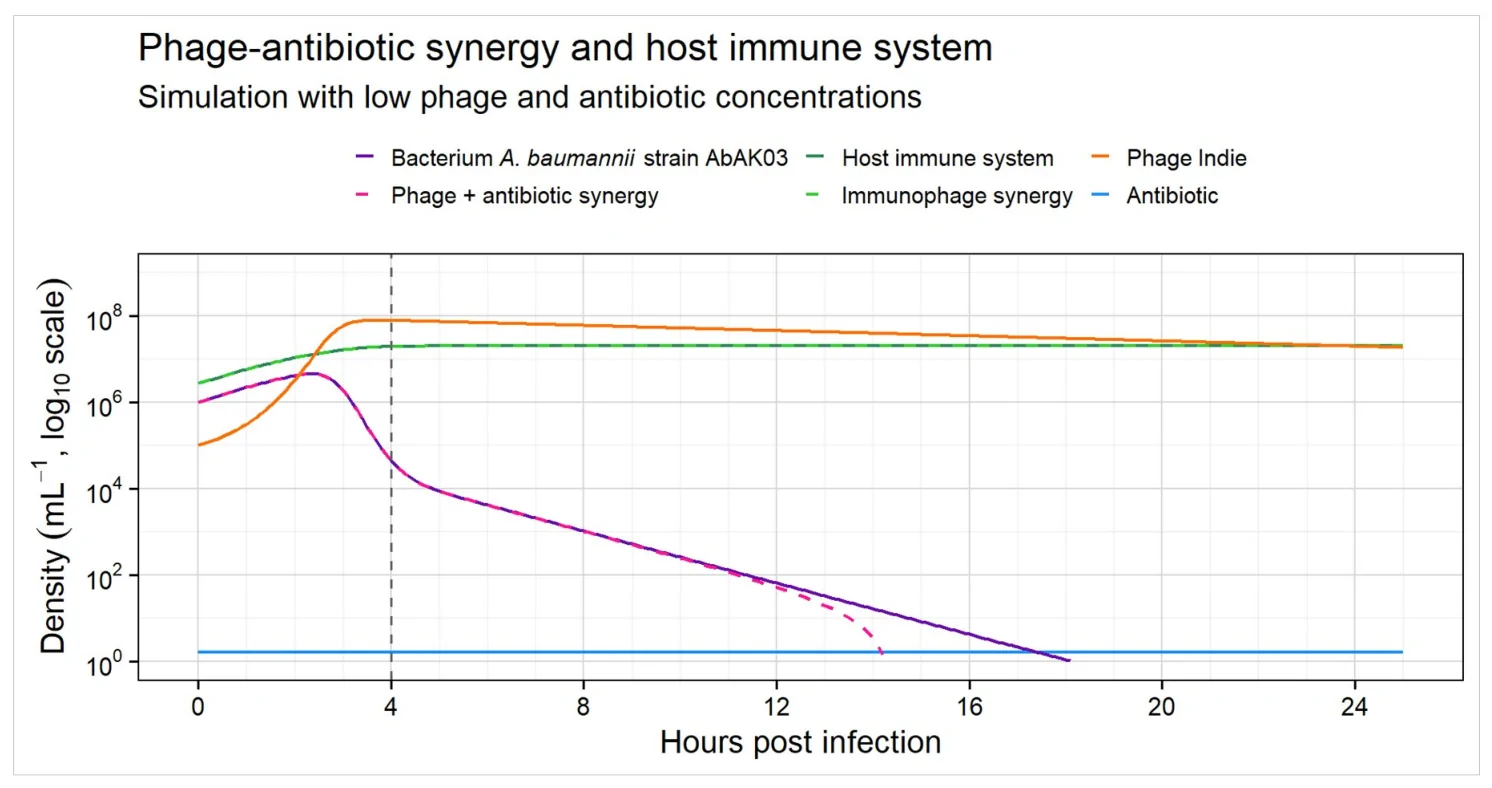
Model 2: Temporal dynamics of bacterial populations, bacteriophages, and host immune system under combined phage–antibiotic therapy. Simulation of infection dynamics over 25 h incorporating phage–antibiotic synergy and phage–host immune system synergy. The bacterial population (purple solid line) increases initially and subsequently declines following phage activity, with the simulation predicting more rapid bacterial clearance under synergistic condition (pink dashed line). The host immune system (green solid line) increases in response to bacterial growth and stabilizes at approximately $10^{7}$ cells mL^−1^ after bacterial reduction. The phage population (orange line) expands during early infection due to replication and decreases as bacterial hosts are depleted. Antibiotic concentration (blue line) remains constant at $A=A_{0}=1.6\;$µg mL^−1^ throughout the simulation and was therefore not dynamically governed by Equation (2). The vertical dashed line at 4 h indicates the onset of the modeled reduction in phage adsorption associated with the population-level reduction in phage susceptibility ($t_{c}$), after which changes in adsorption dynamics and synergy contribute to enhanced bacterial suppression.

The simulations predicted clear divergence between the two scenarios (Figure 3). In the absence of synergy, bacterial density declines gradually, reaching nearly elimination at approximately 18 h. When synergy is included, the model predicts accelerated bacterial clearance, with simulated elimination occurring around 14 h, compared with approximately 18 h in the absence of synergy, consistent with enhanced cooperative killing [20,21,44]. The model predicts that phage amplification contributed substantially to bacterial reduction and was associated with improved overall bacterial clearance dynamics. In contrast, immune response trajectories remained largely unchanged between conditions and stabilized at similar levels, indicating that the improved bacterial control was primarily associated with the phage–antibiotic interaction rather than changes in immune dynamics.

### 2.3. Bacterial Dynamics Under Phage–Antibiotic Synergy with Continuous Antibiotic Infusion and Host Immune System

Model 3 extends the synergistic configuration described in Model 2 by incorporating continuous antibiotic infusion and first-order elimination, allowing evaluation of how sustained antibiotic exposure influences phage–antibiotic synergy under host immune activity.

Unlike Model 2, in which antibiotic concentration was prescribed as a fixed value at $A=A_{0}$, Model 3 represents antibiotic exposure through an explicit infusion–elimination formulation. The antibiotic dynamics are governed by a balance between constant input and first-order elimination, described by a continuous infusion term (input-rate = 0.08 µg mL^−1^ h^−1^) and a first-order elimination rate constant ($\delta_{A}=0.05\;h^{-1}$). The simulation was initialized at $A_{0}=1.6$ µg mL^−1^, which corresponds to the steady-state concentration $q_{A}/\delta_{A}=1.6$ µg mL^−1^. Because the initial antibiotic concentration was set equal to this steady-state value, the infusion rate and elimination rate were balanced throughout the simulation, resulting in a constant antibiotic concentration of 1.6 µg mL^−1^ over the 25 h simulation. Thus, Model 3 explicitly represents the underlying infusion–elimination balance while maintaining sustained antibiotic exposure at the selected steady-state concentration.

Under this formulation, antibiotic exposure is maintained dynamically through the balance between constant infusion and first-order elimination. Because the simulation starts at the steady-state concentration, the antibiotic concentration remains constant at 1.6 µg mL^−1^ throughout the 25 h simulation. Thus, Model 3 differs from Model 2 in that the constant antibiotic exposure is generated by an explicit infusion–elimination balance rather than imposed directly as a fixed concentration.

Bacterial dynamics are therefore shaped by the combined effects of the host immune system, phage replication, and continuously maintained antibiotic pressure. The bacterial population initially exhibits logistic growth but transitions into a sustained decline as phage amplification increases and synergistic interactions remain active throughout the simulation.

**Figure 4:**
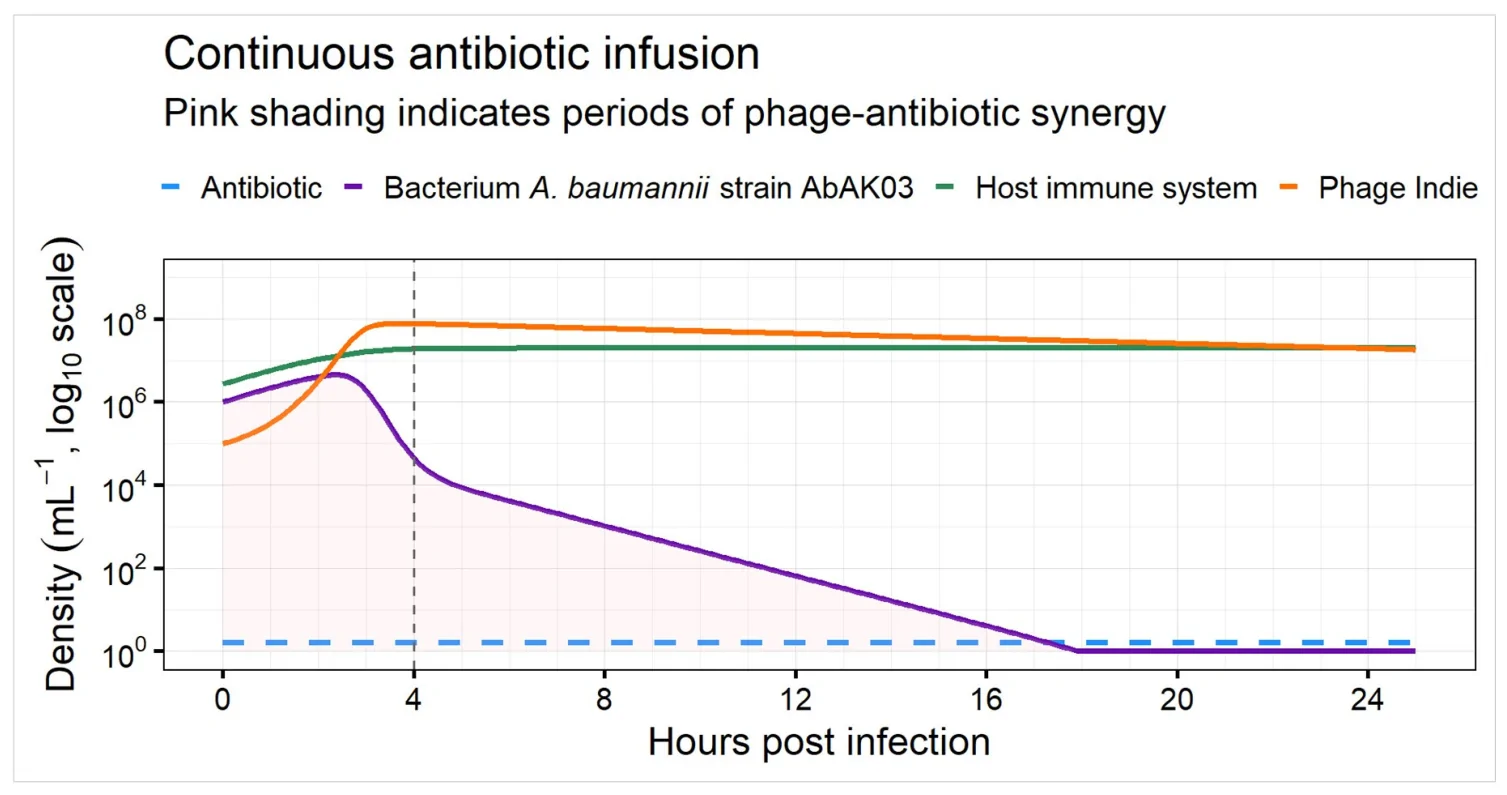
Model 3: Bacterial dynamics under continuous antibiotic infusion and phage–antibiotic synergy. Simulation of bacterial population (*A. baumannii* strain AbAK03, purple solid line), phage Indie (orange solid line), host immune system (neutrophils, green solid line), and antibiotic concentration (ceftazidime, blue dashed line) over 25 h post-infection. Unlike Model 2, in which antibiotic concentration was held constant, Model 3 represents antibiotic exposure through an explicit continuous infusion and first-order elimination balance. Continuous antibiotic exposure was maintained through a balance between constant input and first-order elimination ($\mathrm{input}-\mathrm{rate}=0.08\;µg\;{\mathrm{mL}}^{-1}\;h^{-1}$, $\delta_{A}=0.05\;h^{-1}$), resulting in a constant subinhibitory concentration of 1.6 µg mL^−1^ because the simulation was initialized at the steady-state concentration. The shaded region indicates the period during which the phage–antibiotic synergy term is active ($k_{\mathrm{syn}}=42.5$). The vertical dashed line at 4 h marks the transition time ($t_{c}$) associated with the modeled reduction in phage adsorption.

As a result, the simulation predicted a progressive decrease in bacterial density, reaching the model detection threshold ($10^{0}$ CFU mL^−1^) at approximately 18 h (Figure 4). This result represents a model-derived prediction of bacterial clearance under continuous antibiotic infusion and should not be interpreted as an experimentally demonstrated effect.

### 2.4. Local Sensitivity Analysis

**Figure 5:**
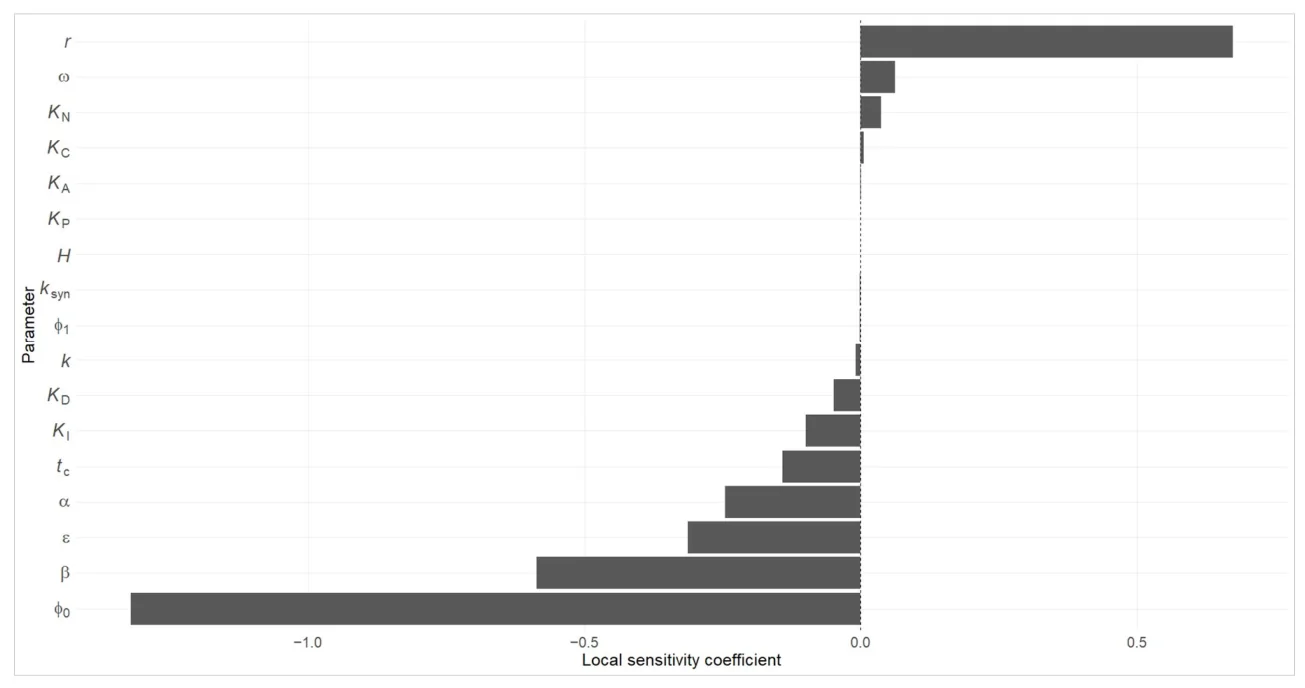
Local sensitivity analysis of bacterial AUC.

The local sensitivity analysis identified a small number of parameters with the greatest influence on bacterial AUC over the 25 h simulation period (Figure 5). The initial phage adsorption coefficient, φ_0_, showed the greatest sensitivity (−1.320), followed by the bacterial growth rate, r(+0.673), and phage burst size, β (−0.586). The bacterial clearance efficiency mediated by the innate immune response, ε (−0.312), and the immune-cell proliferation rate, α (−0.245), were also among the most influential parameters.

Among the parameters associated with the innate immune component, ε showed the greatest influence on bacterial AUC, followed by α, $K_{I}$, $K_{D}$, and $K_{N}$, with sensitivity coefficients of −0.312, −0.245, −0.099, −0.049, and +0.037, respectively. The transition time associated with the population-level reduction in phage susceptibility, $t_{c}$, showed a moderate sensitivity (−0.142), whereas the final adsorption coefficient, φ_1_, had a substantially smaller effect (−0.001). Phage decay, ω, showed a positive sensitivity (+0.062), indicating that increased phage loss was associated with increased bacterial AUC. The remaining parameters showed relatively small local sensitivities under the ±20% perturbations, including k (−0.009), $K_{C}$ (+0.005), $k_{\mathrm{syn}}$ (−0.0006), $K_{A}$(+0.0002), H (−0.0002), and $K_{P}$ (+0.00003).

The primary sensitivity metric was bacterial AUC over the 25 h simulation period. Bacterial density at 25 h (B_25_) was also evaluated as a complementary model output. However, B_25_ reached zero after application of the absorbing boundary under the baseline simulation and remained at zero under both perturbations for most immune-related parameters. Consequently, logarithmic sensitivity coefficients for B_25_ could not be calculated for these parameters and were reported as not applicable. The complete local sensitivity analysis based on bacterial AUC is provided in Supplementary Table S1.

## 3. Discussion

Phage therapy remains an area where significant scientific questions persist, despite growing interest in its clinical potential. In particular, the interactions among bacteria, phages, antibiotics, and the host immune system remain incompletely understood [26]. The present study uses mathematical modeling to examine how the dynamics of phage–bacteria–antibiotic interactions, together with the host immune system, contribute substantially to infection outcomes and to explore the complexity of designing potential therapeutic strategies against multidrug-resistant pathogens such as *Acinetobacter baumannii*. Using a mathematical modeling approach, the predicted influence of phage–host immune system synergy and phage–antibiotic synergy on bacterial clearance was evaluated under different simulated treatment scenarios.

The in silico findings obtained from simulations of phage–antibiotic synergy in the presence of the host immune system, as well as continuous antibiotic infusion under phage treatment, are qualitatively consistent with previous in vitro [19,20,45] and in vivo studies [44]. These studies have reported that phage therapy, particularly when combined with antibiotics and host immune responses, can contribute to bacterial control in experimental settings [46]. Collectively, these observations support the utility of the proposed mathematical model as a computational framework for investigating phage–bacteria–antibiotic–host immune system interactions and for generating hypotheses to guide future preclinical experiments.

Comparison of the three models used highlights the importance of cooperative interactions in determining treatment outcome. Model 1 suggests that neither the host immune system nor antibiotic treatment alone is sufficient to effectively control bacterial proliferation, whereas the inclusion of phages substantially improves bacterial suppression through their capacity for self-amplification within the infection site, consistent with previous studies describing phage–host immune system synergy [23,26]. Model 2 further predicts that incorporation of phage–antibiotic synergy enhances simulated bacterial control beyond that achieved by phage–host immune system synergy alone, supporting the hypothesis that cooperative interactions between antimicrobial agents can overcome limitations associated with individual therapies [20,21]. Model 3 extends these findings by predicting that sustained subinhibitory antibiotic exposure through continuous infusion may prolong the benefits of phage–antibiotic cooperation, thereby creating favorable conditions for bacterial elimination in the model. Together, these results emphasize that successful infection control emerges from the interplay among phages, antibiotics, and the host immune system rather than from the action of any single component alone [18,26].

A major contribution of the present model is the incorporation of a time-dependent phage adsorption rate, φ(t), which captures the progressive reduction in phage infection efficiency associated with the modeled loss of bacterial susceptibility to phage infection through a sigmoidal transition. This formulation provides a phenomenological representation of the population-level reduction in phage susceptibility rather than a mechanistic representation of the molecular or evolutionary processes underlying resistance development. It allows the model to represent the temporal behavior observed experimentally. Biologically, reduced phage adsorption may arise from modification or loss of bacterial surface structures that serve as phage receptors, including changes in components of the bacterial envelope [37]. Such alterations can potentially affect bacterial fitness and may also modify susceptibility to antibiotics [47]. However, the present model does not explicitly represent these molecular mechanisms or their associated fitness consequences because quantitative measurements of growth rate or competitive fitness for phage-resistant derivatives were not available for parameterization. Bacterial populations experiencing reduced phage susceptibility are therefore assumed to retain the same intrinsic growth parameters as the susceptible population. Consequently, the model should be interpreted as evaluating the consequences of reduced phage susceptibility under a neutral fitness-cost assumption rather than predicting the evolutionary fitness of resistant variants.

Under these conditions, the simulations predicted that combined phage–antibiotic therapy would produce a stronger reduction in bacterial density than either phage-only or antibiotic-only treatments, even after the onset of the modeled reduction in phage adsorption. The predicted pattern is qualitatively consistent with previous in vitro studies [20,21], which reported greater reductions in bacterial density following phage–ceftazidime combination therapy than with single-agent treatments and demonstrated that the combined approach can overcome the reduction in phage susceptibility observed approximately 4 h after infection with phage Indie [20]. Importantly, the model does not assume that a population-level reduction in phage susceptibility directly causes antibiotic resensitization. Instead, the population-level reduction in phage susceptibility and phage–antibiotic synergy are represented as distinct population-level processes: reduced phage adsorption captures the temporal change in phage susceptibility, whereas the synergy term represents the experimentally observed enhancement of antibiotic-associated bacterial clearance following phage exposure. Thus, the model does not establish a causal mechanistic pathway linking the reduction in phage susceptibility to antibiotic resensitization.

Another important feature of the model is the explicit incorporation of a phage–antibiotic synergy function, which allows the nonlinear interaction between both agents to be quantitatively evaluated. This formulation predicts that the simulated antibacterial effect increases as phage and antibiotic concentrations exceed specific thresholds, reflecting cooperative effects that have been described experimentally. By incorporating this interaction, the model provides a useful framework for exploring conditions under which combination therapy may eliminate infection even when antibiotics are administered at subinhibitory concentrations. The simulated response is qualitatively consistent with several experimental studies reporting substantial reductions in bacterial density when phage–antibiotic combinations are applied at subinhibitory doses [20,21,48]. Accordingly, mathematical modeling represents an important first step for examining treatment dynamics before undertaking experiments that may be constrained by cost, technical complexity, or ethical considerations [23]. The agreement between model predictions and previously reported experimental observations suggests that computational approaches can be valuable for identifying therapeutic conditions that favor pathogen elimination and for generating hypotheses that can subsequently be tested in vitro and in vivo.

The interpretation of these predictions should also consider parameter uncertainty. Several model parameters were obtained directly or indirectly from experimental observations [20,21,26,27,40], whereas others were estimated from the literature or fitted to previously published biological data. In particular, parameters associated with the population-level reduction in phage susceptibility and phage–antibiotic synergy are subject to uncertainty because they represent population-level effects derived from experimental observations rather than direct measurements of underlying molecular processes. Consequently, the predicted timing and magnitude of bacterial clearance should be interpreted within the assumptions and parameterization of the model. The formal local sensitivity analysis performed in this study further quantified the influence of parameter variation on the simulated bacterial response. The analysis included major model parameters as well as immune-related parameters and showed that parameter perturbations did not affect model predictions uniformly. Parameters associated with phage adsorption, bacterial growth, phage replication, and innate immune activity showed the greatest influence on bacterial AUC, with φ_0_, r, β, ε, and α showing the largest absolute sensitivity coefficients. Among the immune-related parameters, ε and α had the greatest effects, followed by $K_{I}$, $K_{D}$, and $K_{N}$. These findings provide an assessment of the relative robustness of the model predictions and identify parameters whose experimental characterization should be prioritized. Importantly, the sensitivity analysis does not eliminate uncertainty in parameter estimates but indicates which parameters contribute most strongly to variation in the model output under the conditions evaluated.

Several biological limitations should be considered when interpreting the present findings. The model assumes a homogeneous bacterial population and represents host innate immunity primarily through neutrophil dynamics. Consequently, it does not capture spatial heterogeneity, biofilm-associated bacterial populations, phenotypic variation, or intracellular persistence. In particular, *A. baumannii* may survive within host cells, where intracellular bacteria may have reduced exposure to both bacteriophages and antibiotics, potentially influencing treatment efficacy. Incorporating intracellular bacterial compartments, together with spatial structure, biofilm formation, and heterogeneous bacterial phenotypes, would be a valuable direction for future model refinement and could improve the biological and translational relevance of the framework.

In summary, the mathematical models presented here provide a quantitative basis for understanding the complex and nonlinear interactions that emerge when phages, antibiotics, and the host immune system act together against multidrug-resistant *A. baumannii*. By integrating time-dependent phage adsorption, phage–antibiotic synergy, and multiple therapeutic scenarios, the models provide a phenomenological representation of the population-level reduction in phage susceptibility and quantitative insight into how combination therapy may suppress bacterial persistence despite the modeled reduction in phage susceptibility under subinhibitory antibiotic conditions. Taken together, these findings highlight the value of mathematical modeling as a tool for generating hypotheses and informing the design and evaluation of phage–antibiotic therapies prior to experimental and preclinical evaluation.

## 4. Materials and Methods

### Model Simulations

The mathematical model describing infection dynamics was implemented and numerically integrated using R version 4.5.0 (R Core Team, 2025) and the ode solver from the *deSolve* package. Three simulation frameworks were developed:

Model 1 (without synergy): This model described the temporal dynamics of the bacterial population, phage population, antibiotic exposure, and host innate immune system under three treatment conditions: (i) immune system only, (ii) antibiotic treatment combined with the immune system, and (iii) phage therapy combined with the immune system. For the antibiotic-treatment condition, the antibiotic concentration was fixed at $A=A_{0}$ throughout the simulation (dA/dt=0). Thus, the antibiotic concentration was not dynamically updated according to the first-order elimination equation.

Model 2 (with synergy): This model extended Model 1 by incorporating phage–antibiotic synergy together with the phage–host immune system synergy described by [26], allowing the contribution of these interactions to infection control and bacterial clearance to be evaluated. As in the antibiotic-treatment condition of Model 1, the antibiotic concentration in Model 2 was fixed at $A=A_{0}$ throughout the simulation (dA/dt=0), representing constant antibiotic exposure.

Model 3 (with antibiotic infusion): The third model incorporated a continuous antibiotic infusion and first-order elimination, represented by the dynamic equation $dA/dt=q_{A}-\delta_{A}A$, where $q_{A}$ is the constant antibiotic input rate (µg ${mL}^{-1}$ $h^{-1}$) and $\delta_{A}$ is the first-order elimination rate constant ($h^{-1}$). A low-dose continuous infusion regimen was evaluated in the presence of phages and the host immune system. The simulation was initialized at $A_{0}=1.6\;$µg ${mL}^{-1}$, which corresponds to the steady-state concentration $q_{A}/\delta_{A}=1.6$ µg ${mL}^{-1}$, using $q_{A}=0.08$ µg ${mL}^{-1}\;h^{-1}$ and $\delta_{A}=0.05$ $h^{-1}$. Because the initial concentration was set to this steady-state value, dA/dt=0 throughout the simulation and the antibiotic concentration remained constant at 1.6 µg ${mL}^{-1}$, while the infusion-elimination equation was retained explicitly to represent the underlying pharmacokinetic balance.

All simulations were performed over 25 h with a time step of 0.1 h. For graphical representation on a logarithmic scale, non-positive values of bacterial density, phage concentration, and host immune cell density were replaced with a value of 1 in their respective units (1 CFU ${mL}^{-1}$, 1 PFU ${mL}^{-1}$, and 1 cell ${mL}^{-1}$), solely for visualization. Antibiotic concentrations were not subjected to this transformation. The original simulated values were retained for all model calculations and analyses.

All bacterial clearance outcomes reported in the Results represent model-derived simulation predictions unless explicitly identified as experimental observations from previous studies. Experimental data were used for parameterization and model development, but no new experimental validation of the simulated treatment outcomes was performed in the present study.

Simulations were conducted in RStudio (version 2025.05.0 + 496, *Mariposa Orchid*) with Quarto 1.6.42 for reproducible reporting. Data processing and visualization were performed using *ggplot2*, *dplyr*, *tidyr*, and *scales*. The ode function (deSolve) was used for numerical integration and *pivot_longer* (tidyr) for data formatting. Generative artificial intelligence (GenAI) was used to assist with R code development and verification of the mathematical implementation of the model equations. All mathematical formulations, parameter selection, model implementation, and interpretation of results were reviewed and verified by the authors. The complete R scripts used for model implementation and simulations (individual treatments, synergy analysis, and antibiotic infusion scenarios) are provided in Supplementary Information.

Parameters. Parameter values are summarized in Table 1. Bacterial growth, phage infection dynamics, and antibiotic activity parameters were obtained from previous experimental data [20]. Antibiotic exposure was represented differently depending on the simulation framework, as described above. Parameters describing the host immune system were fitted using neutrophil recruitment data from murine acute lung injury models [26,34].

Parameters associated with phage–antibiotic synergy and the population-level reduction in phage susceptibility, including the maximum synergy magnitude ($k_{syn}$), the antibiotic synergy constant ($K_{A}$), the phage synergy constant ($K_{P}$), and the characteristic time associated with the experimentally observed reduction in phage susceptibility ($t_{c}$), were estimated from previous experimental measurements [20]. Experimental observations demonstrated that *A. baumannii* strain AbAK03 was resistant to ceftazidime and that reduced phage susceptibility was observed approximately 4 h post-infection [20]. These observations were used to parameterize antibiotic activity and the transition time ($t_{c}$) governing the decline in phage adsorption efficiency.

The time-dependent reduction in phage adsorption was used as a phenomenological representation of the population-level reduction in phage susceptibility. The model does not explicitly represent the genetic emergence, selection, or expansion of resistant mutants, nor does it explicitly incorporate molecular changes in phage receptors or other bacterial surface structures. Accordingly, t_c_ represents the characteristic transition time associated with the experimentally observed reduction in phage susceptibility rather than the time at which a specific resistant genotype is assumed to arise.

The model also does not explicitly incorporate a fitness cost associated with phage resistance. Resistant bacteria are therefore assumed to retain the same intrinsic growth parameters as the susceptible bacterial population. This assumption was adopted because quantitative measurements of growth rate or competitive fitness for phage-resistant derivatives were not available for parameterization. Consequently, the model evaluates the effects of reduced phage susceptibility under a neutral fitness-cost assumption rather than predicting the evolutionary fitness of resistant variants.

The phage–antibiotic synergy term and the time-dependent phage adsorption function were parameterized as separate model components. Thus, the model does not assume that a population-level reduction in phage susceptibility directly causes antibiotic resensitization. The synergy term represents the experimentally observed enhancement of antibiotic-associated bacterial clearance following phage exposure, whereas the time-dependent adsorption function represents the population-level reduction in phage susceptibility.

Individual drug effects were modeled using Hill-type functions. Antibiotic inhibition was expressed as:

(7) $$\frac{A}{A+K_{A}}$$

and phage-response function as:

(8) $$\frac{P}{P+K_{P}}$$

Phage adsorption dynamics were defined by a time-dependent function in which $\varphi_{0}$ represents the initial adsorption rate at time zero, $\varphi_{1}$ is the adsorption rate after the modeled reduction in phage susceptibility, $t_{c}$ denotes the transition time at which φ reaches the midpoint between $\varphi_{0}$ and $\varphi_{1}$, and k defines the slope of the sigmoidal transition, reflecting how rapidly φ decreases over time.

Local sensitivity analysis. A local sensitivity analysis was performed to quantify the influence of model parameters on the predicted treatment dynamics. Seventeen parameters representing bacterial growth, phage–host interactions, phage decay, innate immune activity, population-level reduction in phage susceptibility, and phage–antibiotic synergy were evaluated individually. Each parameter was perturbed by −20% and +20% relative to its baseline value while all other parameters were kept unchanged.

The bacterial area under the curve over the 25 h simulation period (AUC_B_) was used as the primary sensitivity endpoint, as it captures the cumulative bacterial burden throughout the treatment period. Bacterial density at 25 h (B_25_), phage density at 25 h (P_25_), and immune-cell density at 25 h (I_25_) were evaluated as secondary endpoints.

For each parameter p, the local sensitivity coefficient was calculated as follows:

(9) $$S_{p=\frac{In(Y_{+20\%}/Y_{-20\%})}{In(1.2/0.8)}}$$

where $Y_{+20\%}$ and $Y_{-20\%}$ represent the model output obtained after increasing or decreasing parameter p by 20%, respectively. Positive sensitivity coefficients indicate that an increase in the parameter is associated with an increase in the model output, whereas negative coefficients indicate an inverse relationship. Parameters were ranked according to the absolute value of their sensitivity coefficient.

Bacterial AUC was calculated as:

(10) $${AUC}_{B}=\int_{0}^{25}B\;(t)dt$$

where $B\left(t\right)$ represents bacterial density at time t. Because bacterial extinction can occur during the simulations, an absorbing boundary at B = 0 was applied once the bacterial population reached 10^−8^ CFU mL^−1^. Following this point, bacterial density was maintained at zero for the remainder of the simulation. This numerical treatment prevented nonphysical negative bacterial densities while preserving the simulated trajectory before extinction.

For Model 2, antibiotic concentration was maintained at the fixed initial concentration (A = A_0_); therefore, the antibiotic elimination parameter, δ_A_, was not included in the sensitivity analysis because it is inactive under this formulation. The sensitivity analysis was performed using the same parameterization and simulation conditions described above. Complete sensitivity results are provided in the Supplementary Material.

Model assumptions and limitations. The model assumes logistic bacterial growth, deterministic phage–bacteria interactions, and a simplified innate immune response represented by neutrophils. It also assumes a homogeneous bacterial population and does not explicitly represent bacterial phenotypic heterogeneity or subpopulations with distinct susceptibility to phages or antibiotics. Population-level reduction in phage susceptibility is represented phenomenologically through a time-dependent reduction in phage adsorption, while phage–antibiotic synergy is modeled using a saturable Hill-type function rather than explicit molecular mechanisms. The model does not explicitly account for stochastic effects, spatial heterogeneity, biofilm formation, or intracellular bacterial persistence. In particular, *A. baumannii* may persist within host cells, where intracellular bacteria may have reduced exposure to both bacteriophages and antibiotics; this process could therefore influence treatment efficacy but is not represented in the present framework. Incorporating spatial structure, biofilm-associated populations, phenotypic heterogeneity, and intracellular bacterial compartments would be potential directions for future model refinement and could improve its biological and translational relevance. Therefore, the simulations are intended to explore treatment dynamics and generate experimentally testable hypotheses rather than directly predict clinical outcomes.

## 5. Conclusions

The mathematical models developed in this study provide quantitative insight into phage–bacteria–antibiotic–host immune system interactions during simulated treatment of multidrug-resistant *A. baumannii*. The incorporation of a time-dependent population-level reduction in phage susceptibility, phage–antibiotic synergy, and the host immune system allowed the model to simulate different bacterial clearance dynamics under combined treatment scenarios. The formal local sensitivity analysis further identified the relative influence of major and immune-related parameters on model predictions, providing information on parameter robustness and priorities for future experimental validation. Overall, the model suggests that combination therapy may represent a promising strategy under the simulated conditions and provides a computational framework for generating hypotheses and informing future experimental and preclinical studies.

## Data Availability

The R scripts used for simulating the combined phage–antibiotic therapy model and producing the primary figures are publicly accessible in the Supplementary Information.

## Supplementary Materials

The following supporting information can be downloaded at: https://www.mdpi.com/article/10.3390/antibiotics15090919/s1, Table S1: Complete local sensitivity analysis of Model 2; Code S1: R code for bacterial dynamics under individual treatments; Code S2: R code for phage-antibiotic synergy and host immune system; Code S3: R code for continuous antibiotic infusion; Code S4: R code for formal local sensitivity analysis.
