# Supplementary material for: Modeling Phage–Antibiotic Synergy, Innate Immunity, and Phage Resistance in Multidrug-Resistant Acinetobacter baumannii
Source: Antibiotics (Basel). 2026 Sep 17;15(9):919. doi: 10.3390/antibiotics15090919 (PMC13603412; doi:10.3390/antibiotics15090919)
Supplement: Supplementary file 1 [file antibiotics-15-00919-s001.zip › antibiotics-4527836-supplementary.pdf]

Modeling phage-antibiotic synergy, innate immunity, and phage resistance in multidrug-resistant *Acinetobacter baumannii*.

Code S1 R Code for Bacterial dynamics under individual treatments.

```
# ------------------------------
# Required libraries
# ------------------------------
library(deSolve)
library(ggplot2)
library(tidyr)
library(dplyr)
library(scales)
library(grid)


# ------------------------------
# Model definition
# ------------------------------
infection_model_individual <- function(time, state, parameters) {

  with(as.list(c(state, parameters)), {

    phi_t <- phi1 + (phi0 - phi1) / (1 + exp(k * (time - tc)))

    synergy <- 0

    dB <- r * B * (1 - B / K_C) -
      phi_t * B * P -
      (epsilon * I * B) / (1 + B / K_D) -
```

```
    synergy

   dP <- beta * phi_t * B * P - omega * P

   dI <- alpha * I * (1 - I / K_I) *
    (B / (B + K_N))

   dA <- 0

   list(c(dB, dP, dI, dA))
  })
}

# -------------------------------
# Model parameters
# -------------------------------
parameters <- c(
  r = 1,
  K_C = 1e8,
  beta = 13,
  phi0 = 6e-8,
  phi1 = 8e-10,
  omega = 0.069,
  alpha = 0.97,
  epsilon = 8.2e-8,
  K_D = 2.2e6,
  K_I = 2.4e7,
```

```
  K_N = 1e5,
  k_syn = 0,
  K_A = 5.81,
  K_P = 1e6,
  tc = 4,
  k = 3,
  H = 1
)

# ------------------------------
# Initial conditions
# ------------------------------
state_base <- c(
  B = 1e6,
  P = 1e7,
  I = 2.7e6,
  A = 10
)

times <- seq(0, 25, by = 0.1)

# ------------------------------
# Treatment scenarios
# ------------------------------
state_phage <- state_base

state_antibiotic <- state_base
```

```
state_antibiotic["P"] <- 0

state_immunity <- state_base
state_immunity["P"] <- 0
state_immunity["A"] <- 0

# ------------------------------
# Simulations
# ------------------------------
out_phage <- ode(y = state_phage, times = times,
                 func = infection_model_individual, parms = parameters)

out_antibiotic <- ode(y = state_antibiotic, times = times,
                      func = infection_model_individual, parms = parameters)

out_immunity <- ode(y = state_immunity, times = times,
                    func = infection_model_individual, parms = parameters)

# ------------------------------
# Data preparation
# ------------------------------
df_phage <- as.data.frame(out_phage) %>%
  mutate(Treatment = "Phage")

df_antibiotic <- as.data.frame(out_antibiotic) %>%
  mutate(Treatment = "Antibiotic")
```

```
df_immunity <- as.data.frame(out_immunity) %>%
  mutate(Treatment = "Immunity")

df_all <- bind_rows(df_phage, df_antibiotic, df_immunity)

df_long <- pivot_longer(df_all,
                        cols = c(B, P, I, A),
                        names_to = "Variable",
                        values_to = "Value")

# -------------------------------
# Colors
# -------------------------------
colors <- c(
  B = "#6A0DAD",
  P = "#FF6F00",
  I = "#2E8B57",
  A = "#1E90FF"
)

# -------------------------------
# Plot function
# -------------------------------
make_plot <- function(trt) {

  vars_to_show <- switch(
    trt,
```

```
  "Immunity" = c("B", "I"),
  "Antibiotic" = c("B", "A", "I"),
  "Phage" = c("B", "P", "I")
)

ggplot(
  filter(df_long,
         Treatment == trt,
         Variable %in% vars_to_show),
  aes(x = time, y = Value, color = Variable)
) +

  geom_line(linewidth = 1.8) +

  scale_color_manual(
    values = colors,
    breaks = vars_to_show,
    labels = c(
      B = expression("Bacterium "*italic("A. baumannii")*" strain AbAK03"),
      P = "Phage Indie",
      I = "Host immune system",
      A = "Antibiotic"
    )[vars_to_show]
  ) +

  scale_y_log10(
    labels = trans_format("log10", math_format(10^.x)),
```

```
  limits = c(1e0, 1e9),
  breaks = trans_breaks("log10", function(x) 10^x)
) +

scale_x_continuous(
  limits = c(0, 25),
  breaks = seq(0, 24, by = 4),
  minor_breaks = seq(0, 24, by = 2)
) +

geom_vline(xintercept = 4,
           linetype = "dashed",
           color = "black",
           linewidth = 1) +

labs(
  title = switch(
    trt,
    "Immunity" = "Host immune system only",
    "Antibiotic" = "Antibiotic and host immune system",
    "Phage" = "Phage and host immune system"
  ),
  x = "Hours post infection",
  y = expression(Density~(mL^{-1}*","~log[10]~scale))
) +

theme_minimal(base_size = 25) +
```

```
theme(
  legend.position = "top",
  legend.title = element_blank(),

  panel.border = element_rect(
    colour = "black",
    fill = NA,
    linewidth = 1
  ),

  panel.grid.major = element_line(
    colour = "grey75",
    linewidth = 0.5
  ),

  panel.grid.minor = element_line(
    colour = "grey90",
    linewidth = 0.3
  ),

  axis.ticks = element_line(
    colour = "black",
    linewidth = 1.2
  ),

  axis.ticks.length = unit(6, "pt"),
```

```
      axis.text = element_text(
        colour = "black",
        size = 22
      ),

      axis.title = element_text(
        colour = "black",
        size = 24
      ),

      plot.title = element_text(
        hjust = 0,
        face = "plain",
        size = 24
      )
    )
}

# -------------------------------
# Generate plots
# -------------------------------
pA <- make_plot("Immunity")
pB <- make_plot("Antibiotic")
pC <- make_plot("Phage")

# -------------------------------
```

```
# Show plots
# -------------------------------
pA
pB
pC

# -------------------------------
# Save figures
# -------------------------------
ggsave("Figure2A_Immunity.png", pA, width = 10, height = 7, dpi = 600)
ggsave("Figure2B_Antibiotic.png", pB, width = 10, height = 7, dpi = 600)
ggsave("Figure2C_Phage.png", pC, width = 10, height = 7, dpi = 600)
```

Code S2 R Code for phage-antibiotic synergy and host immune system

```r
# -------------------------------
# Required libraries
# -------------------------------
library(deSolve)
library(ggplot2)
library(tidyr)
library(scales)
library(dplyr)

# -------------------------------
# Infection dynamics model
# -------------------------------
infection_model <- function(time, state, parameters) {
  with(as.list(c(state, parameters)), {

    phi_t <- phi1 + (phi0 - phi1) / (1 + exp(k * (time - tc)))

    synergy <- k_syn * (
      (A^H / (A^H + K_A^H)) *
      (P / (P + K_P))
    )

    dB <- r * B * (1 - B / K_C) -
      phi_t * B * P -
      (epsilon * I * B) / (1 + B / K_D) -
      synergy
```

```
    dP <- beta * phi_t * B * P - omega * P

    dI <- alpha * I * (1 - I / K_I) *
      (B / (B + K_N))

    dA <- 0

    return(list(c(dB, dP, dI, dA)))
  })
}

# -------------------------------
# Parameters
# -------------------------------
parameters_base <- c(
  r = 1,
  K_C = 1e8,
  beta = 13,
  phi0 = 6e-8,
  phi1 = 8e-10,
  omega = 0.069,
  alpha = 0.97,
  epsilon = 8.2e-8,
  K_D = 2.2e6,
  K_I = 2.4e7,
  K_N = 1e5,
```

```
  tc = 4,
  k = 3,
  H = 1
)

parameters_no_syn <- c(parameters_base, k_syn = 0, K_A = 1, K_P = 1)
parameters_syn    <- c(parameters_base, k_syn = 42.5, K_A = 5.81, K_P = 1e6)

# ------------------------------
# Initial conditions
# ------------------------------
state <- c(B = 1e6, P = 1e5, I = 2.7e6, A = 1.6)
times <- seq(0, 25, by = 0.1)

# ------------------------------
# Simulations
# ------------------------------
out_no_syn <- ode(y = state, times = times,
                  func = infection_model, parms = parameters_no_syn)

out_syn <- ode(y = state, times = times,
               func = infection_model, parms = parameters_syn)

df_no_syn <- as.data.frame(out_no_syn) %>% mutate(Synergy = "No")
df_syn    <- as.data.frame(out_syn) %>% mutate(Synergy = "Yes")

df_all <- bind_rows(df_no_syn, df_syn)
```

```
# ------------------------------
# Reshape data
# ------------------------------
df_long <- pivot_longer(
  df_all,
  cols = c("B", "P", "I", "A"),
  names_to = "Variable",
  values_to = "Value"
)

df_long <- df_long %>%
  mutate(
    Var_Syn = case_when(
      Variable == "B" & Synergy == "Yes" ~ "Bacterial synergy",
      Variable == "B" & Synergy == "No"  ~ "Bacterial no synergy",
      Variable == "I" & Synergy == "Yes" ~ "Host immunity synergy",
      Variable == "I" & Synergy == "No"  ~ "Host immunity no synergy",
      Variable == "P" ~ "Phage",
      Variable == "A" ~ "Antibiotic"
    )
  )

# ------------------------------
# Colors
# ------------------------------
colors <- c(
```

```r
  "Bacterial no synergy" = "#6A0DAD",
  "Bacterial synergy" = "#FF1493",
  "Host immunity no synergy" = "#2E8B57",
  "Host immunity synergy" = "#32CD32",
  "Phage" = "#FF6F00",
  "Antibiotic" = "#1E90FF"
)

# ------------------------------
# Line types
# ------------------------------
linetypes <- c(
  "Bacterial no synergy" = "solid",
  "Bacterial synergy" = "dashed",
  "Host immunity no synergy" = "solid",
  "Host immunity synergy" = "dashed",
  "Phage" = "solid",
  "Antibiotic" = "solid"
)

# ------------------------------
# Labels (A. baumannii italic)
# ------------------------------
labels <- c(
  "Bacterial no synergy" =
    expression("Bacterium " * italic("A. baumannii") * " strain AbAK03"),
  "Bacterial synergy" = "Phage + antibiotic synergy",
```

```
  "Host immunity no synergy" = "Host immune system",
  "Host immunity synergy" = "Immunophage synergy",
  "Phage" = "Phage Indie",
  "Antibiotic" = "Antibiotic"
)

# ------------------------------
# Plot
# ------------------------------
ggplot(df_long, aes(x = time, y = Value,
                    color = Var_Syn,
                    linetype = Var_Syn)) +

  geom_line(size = 1.3) +

  scale_color_manual(values = colors,
                     breaks = names(linetypes),
                     labels = labels) +

  scale_linetype_manual(values = linetypes,
                        breaks = names(linetypes),
                        labels = labels) +

  scale_y_log10(
    labels = trans_format("log10", math_format(10^.x)),
    limits = c(1, 1e9),
    breaks = trans_breaks("log10", function(x) 10^x)
```

```
) +

# ------------------------------
# X-axis MORE TICKS (FIX HERE)
# ------------------------------
scale_x_continuous(
  limits = c(0, 25),
  breaks = seq(0, 25, by = 4),   # más etiquetas
  minor_breaks = seq(0, 25, by = 1)
) +

geom_vline(xintercept = 4,
           linetype = "dashed",
           color = "grey40",
           size = 1) +

labs(
  title = "Phage-antibiotic synergy and host immune system",
  subtitle = "Simulation with low phage and antibiotic concentrations",
  x = "Hours post infection",
  y = "Density (mL⁻¹, log₁₀ scale)"
) +

# ------------------------------
# Theme (GRID ADDED HERE)
# ------------------------------
theme_minimal(base_size = 25) +
```

```r
theme(

  legend.position = "top",
  legend.box = "horizontal",
  legend.title = element_blank(),
  legend.text = element_text(size = 18),

  panel.border = element_rect(color = "black", fill = NA, size = 1),

  axis.ticks = element_line(size = 1),
  axis.ticks.length = unit(7, "pt"),
  axis.text = element_text(color = "black"),

  # ✓ GRID RESTORED
  panel.grid.major = element_line(color = "grey85", linewidth = 0.6),
  panel.grid.minor = element_line(color = "grey92", linewidth = 0.3)
)
```

Code S3 R Code for Continuous antibiotic infusion

```
# ------------------------------
# Required libraries
# ------------------------------
library(deSolve)
library(ggplot2)
library(tidyr)
library(scales)
library(dplyr)
library(grid)

# ------------------------------
# Infection model with antibiotic
# ------------------------------
infection_model_infusion <- function(time, state, parameters) {

  with(as.list(c(state, parameters)), {

    phi_t <- phi1 + (phi0 - phi1) /
      (1 + exp(k * (time - tc)))

    synergy <- k_syn * (
      (A^H / (A^H + K_A^H)) *
      (P / (P + K_P))
    )

    dB <- r * B * (1 - B / K_C) -
```

```
      phi_t * B * P -
      (epsilon * I * B) / (1 + B / K_D) -
      synergy

    dP <- beta * phi_t * B * P -
      omega * P

    dI <- alpha * I *
      (1 - I / K_I) *
      (B / (B + K_N))

    dA <- input_rate -
      delta_A * A

    return(list(c(dB, dP, dI, dA)))
  })
}

# ------------------------------
# Base parameters
# ------------------------------
parameters_base <- c(
  r = 1,
  K_C = 1e8,
  beta = 13,
  phi0 = 6e-8,
  phi1 = 8e-10,
```

```
  omega = 0.069,
  alpha = 0.97,
  epsilon = 8.2e-8,
  K_D = 2.2e6,
  K_I = 2.4e7,
  K_N = 1e5,
  k_syn = 0.5,
  K_A = 5.81,
  K_P = 1e6,
  tc = 4,
  k = 3,
  H = 1,
  delta_A = 0.05
)

# ------------------------------
# Initial states
# ------------------------------
state_base <- c(
  B = 1e6,
  P = 1e5,
  I = 2.7e6,
  A = 0
)

# ------------------------------
# Simulation time
```

```
# ------------------------------
times <- seq(0, 25, by = 0.1)

# ------------------------------
# Single scenario: Continuous infusion
# ------------------------------
parameters <- c(
  parameters_base,
  input_rate = 0.08,
  A = 1.6
)

# set initial antibiotic concentration
state <- state_base
state["A"] <- parameters["A"]

# ------------------------------
# Run simulation
# ------------------------------
out <- ode(
  y = state,
  times = times,
  func = infection_model_infusion,
  parms = parameters
)

df <- as.data.frame(out)
```

```
# ------------------------------
# Long format
# ------------------------------
df_long <- pivot_longer(
  df,
  cols = c(B, P, I, A),
  names_to = "Variable",
  values_to = "Value"
) %>%
  mutate(
    Value = pmax(Value, 1),
    Synergy_active = Variable == "B"
  )

# ------------------------------
# Colors
# ------------------------------
colors <- c(
  B = "#6A0DAD",
  P = "#FF6F00",
  I = "#2E8B57",
  A = "#1E90FF"
)

# ------------------------------
# Labels (with italic A. baumannii)
```

```r
# -------------------------------
labels <- c(
  B = expression(paste("Bacterium ", italic("A. baumannii"), " strain AbAK03")),
  P = "Phage Indie",
  I = "Host immune system",
  A = "Antibiotic"
)

# -------------------------------
# Plot
# -------------------------------
p <- ggplot(
  df_long,
  aes(
    x = time,
    y = Value,
    color = Variable,
    linetype = Variable
  )
) +

  geom_line(linewidth = 2) +

  geom_ribbon(
    data = subset(df_long, Synergy_active),
    aes(
      x = time,
```

```
    ymin = 1,
    ymax = Value
  ),
  fill = "pink",
  alpha = 0.15,
  inherit.aes = FALSE
) +

scale_color_manual(values = colors, labels = labels) +

scale_linetype_manual(
  values = c(B = "solid", P = "solid", I = "solid", A = "dashed"),
  labels = labels
) +

scale_y_log10(
  labels = trans_format("log10", math_format(10^.x)),
  limits = c(1, 1e9),
  breaks = c(1, 1e2, 1e4, 1e6, 1e8)
) +

scale_x_continuous(
  limits = c(0, 25),
  breaks = seq(0, 24, by = 4),
  minor_breaks = seq(0, 25, by = 1)
) +
```

```
geom_vline(
  xintercept = 4,
  linetype = "dashed",
  color = "grey40",
  linewidth = 1
) +

labs(
  title = "Continuous antibiotic infusion",
  subtitle = "Pink shading indicates periods of phage-antibiotic synergy",
  x = "Hours post infection",
  y = expression("Density (mL⁻¹, log₁₀ scale)")
) +

theme_minimal(base_size = 30) +

theme(
  legend.position = "top",
  legend.title = element_blank(),
  panel.border = element_rect(color = "black", fill = NA, linewidth = 1),
  axis.ticks = element_line(color = "black", linewidth = 1.5),
  axis.ticks.length = unit(7, "pt"),
  axis.text = element_text(color = "black"),
  panel.grid.major = element_line(color = "grey85", linewidth = 0.5),
  panel.grid.minor = element_line(color = "grey92", linewidth = 0.3)
)
```

```
print(p)

# -------------------------------
# Save figure
# -------------------------------
ggsave(
  "Continuous_antibiotic_infusion.png",
  plot = p,
  width = 12,
  height = 8,
  dpi = 600
)
```

# Code S4 FORMAL LOCAL SENSITIVITY ANALYSIS

# Model 2: Phage-antibiotic synergy + innate immunity

# *Acinetobacter baumannii*

```
library(deSolve)

library(dplyr)

library(tidyr)

library(ggplot2)



# ============================================================

# BASELINE PARAMETERS

# ============================================================


parms_base <- c(

  r = 1,

  K_C = 1e8,

  beta = 13,

  phi0 = 6e-8,

  phi1 = 8e-10,

  omega = 0.069,

  alpha = 0.97,

  epsilon = 8.2e-8,

  K_D = 2.2e6,

  K_I = 2.4e7,

  K_N = 1e5,

  k_syn = 42.5,
```

```
  K_A = 5.81,
  K_P = 1e6,
  H = 1,
  tc = 4,
  k = 3,
  A0 = 10,
  delta_A = 0.05
)

# ============================================================
# INITIAL CONDITIONS
# ============================================================

state0 <- c(
  B = 1e6,
  P = 1e7,
  I = 2.7e6
)

# ============================================================
# SIMULATION TIME
# ============================================================

times <- seq(
  0,
```

```
  25,
  by = 0.1
)


# ============================================================
# PARAMETERS INCLUDED IN SENSITIVITY ANALYSIS
# ============================================================

sensitivity_parameters <- c(
  "r",
  "K_C",
  "beta",
  "phi0",
  "phi1",
  "omega",
  "alpha",
  "epsilon",
  "K_D",
  "K_I",
  "K_N",
  "k_syn",
  "K_A",
  "K_P",
  "H",
  "tc",
  "k"
```

```
)


# ============================================================
# MODEL 2
# ============================================================

model <- function(t, state, parms) {

  with(as.list(c(state, parms)), {

    # Time-dependent phage adsorption
    phi_t <- phi1 +
      (phi0 - phi1) /
      (1 + exp(k * (t - tc)))



    # Constant antibiotic concentration
    A <- A0



    # Immune response
    dI <- alpha *
      (B / (B + K_N)) *
      (1 - I / K_I) *
      I
```

```
# Phage dynamics
dP <- beta *
  phi_t *
  B *
  P -
  omega *
  P


# Phage-antibiotic synergy
synergy <- k_syn *
  (A^H / (A^H + K_A^H)) *
  (P^H / (P^H + K_P^H))


# Bacterial dynamics
dB <- r *
  B *
  (1 - B / K_C) -
  phi_t *
  B *
  P -
  (epsilon * I * B /
     (1 + B / K_D)) -
  synergy
```

```
    list(
      c(
        dB,
        dP,
        dI
      )
    )
  })
}


# ============================================================
# AREA UNDER THE CURVE
# ============================================================

calc_auc <- function(time, value) {

  sum(
    diff(time) *
      (
        head(value, -1) +
          tail(value, -1)
      ) / 2
  )
}
```

```
# ============================================================
# MODEL SIMULATION FUNCTION
# ============================================================

run_model <- function(parms) {

  B_threshold <- 1e-8


  # Root function for bacterial extinction
  root_B <- function(t, state, parms) {
    state["B"] - B_threshold
  }


  # Event function after bacterial extinction
  event_B <- function(t, state, parms) {
    state["B"] <- 0
    state
  }


  # Solve ODE system
  out <- ode(
    y = state0,
    times = times,
```

```
  func = model,
  parms = parms,
  method = "lsodar",
  rootfun = root_B,
  events = list(
    func = event_B,
    root = TRUE,
    terminalroot = 1
  ),
  rtol = 1e-8,
  atol = 1e-10,
  maxsteps = 100000
)

out <- as.data.frame(out)

# Last simulated time
last_time <- tail(
  out$time,
  1
)

# Extend simulation after bacterial extinction
if (
```

```
  last_time < max(times) &&
  tail(out$B, 1) <= B_threshold
) {

  t_ext <- last_time

  P_ext <- max(
    tail(out$P, 1),
    0
  )

  I_ext <- max(
    tail(out$I, 1),
    0
  )


  post_times <- times[
    times > t_ext
  ]


  if (
    length(post_times) > 0
  ) {

    post <- data.frame(
```

```
      time = post_times,
      B = 0,
      P = P_ext *
        exp(
          -parms["omega"] *
            (post_times - t_ext)
        ),
      I = I_ext
    )


    out <- bind_rows(
      out,
      post
    )
  }
}


# Remove numerical artifacts
out$B[
  out$B <= B_threshold
] <- 0

out$P[
  out$P < 0
] <- 0
```

```
  out$I[
    out$I < 0
  ] <- 0


  # Keep only requested simulation time
  out <- out[
    out$time <= max(times),
  ]


  # Sort by time
  out <- out[
    order(out$time),
  ]


  # Remove duplicated time points
  out <- out[
    !duplicated(out$time),
  ]


  return(out)
}
```

```
# ============================================================
# BASELINE SIMULATION
# ============================================================

baseline <- run_model(
  parms_base
)


if (
  max(baseline$time) < 25
) {

  stop(
    "Baseline simulation did not reach 25 h."
  )
}


# ============================================================
# BASELINE OUTCOMES
# ============================================================

baseline_AUC_B <- calc_auc(
  baseline$time,
  baseline$B
```

```
)

baseline_B25 <- tail(
  baseline$B,
  1
)

baseline_P25 <- tail(
  baseline$P,
  1
)

baseline_I25 <- tail(
  baseline$I,
  1
)

# ============================================================
# SENSITIVITY CALCULATION
# ============================================================

log_parameter_change <- log(
  1.2 / 0.8
```

```
)

sensitivity_results <- data.frame()

for (
  p in sensitivity_parameters
) {

  # -20%
  parms_minus <- parms_base

  parms_minus[p] <-
    parms_base[p] * 0.80

  # +20%
  parms_plus <- parms_base

  parms_plus[p] <-
    parms_base[p] * 1.20

  # Simulations
  sim_minus <- run_model(
```

```
  parms_minus
)

sim_plus <- run_model(
  parms_plus
)


# Check simulation length
if (
  max(sim_minus$time) < 25 ||
  max(sim_plus$time) < 25
) {

  stop(
    paste(
      "A sensitivity simulation did not reach 25 h for:",
      p
    )
  )
}


# Check finite values
if (
  any(!is.finite(sim_minus$B)) ||
  any(!is.finite(sim_plus$B)) ||
```

```
  any(!is.finite(sim_minus$P)) ||
  any(!is.finite(sim_plus$P)) ||
  any(!is.finite(sim_minus$I)) ||
  any(!is.finite(sim_plus$I))
) {

  stop(
    paste(
      "Non-finite values detected for:",
      p
    )
  )
}


# AUC
AUC_minus <- calc_auc(
  sim_minus$time,
  sim_minus$B
)

AUC_plus <- calc_auc(
  sim_plus$time,
  sim_plus$B
)
```

```
)

# Final values
B25_minus <- max(
  tail(sim_minus$B, 1),
  0
)

B25_plus <- max(
  tail(sim_plus$B, 1),
  0
)


P25_minus <- max(
  tail(sim_minus$P, 1),
  0
)

P25_plus <- max(
  tail(sim_plus$P, 1),
  0
)


I25_minus <- max(
  tail(sim_minus$I, 1),
  0
)
```

```
I25_plus <- max(
  tail(sim_plus$I, 1),
  0
)


# Sensitivity coefficient for bacterial AUC
S_AUC <- if (
  AUC_minus > 0 &&
  AUC_plus > 0
) {

  log(
    AUC_plus / AUC_minus
  ) /
    log_parameter_change

} else {

  NA_real_
}


# Sensitivity coefficient for B25
S_B25 <- if (
  B25_minus > 0 &&
```

```
  B25_plus > 0
) {

  log(
    B25_plus / B25_minus
  ) /
    log_parameter_change

} else {

  NA_real_
}


# Sensitivity coefficient for P25
S_P25 <- if (
  P25_minus > 0 &&
  P25_plus > 0
) {

  log(
    P25_plus / P25_minus
  ) /
    log_parameter_change

} else {
```

```
  NA_real_
}


# Sensitivity coefficient for I25
S_I25 <- if (
  I25_minus > 0 &&
  I25_plus > 0
) {

  log(
    I25_plus / I25_minus
  ) /
    log_parameter_change

} else {

  NA_real_
}


# Store results
sensitivity_results <- rbind(
  sensitivity_results,

  data.frame(
    Parameter = p,
```

```
      Baseline = parms_base[p],

      AUC_B_minus20 = AUC_minus,
      AUC_B_plus20 = AUC_plus,
      Sensitivity_AUC_B = S_AUC,

      B25_minus20 = B25_minus,
      B25_plus20 = B25_plus,
      Sensitivity_B25 = S_B25,

      P25_minus20 = P25_minus,
      P25_plus20 = P25_plus,
      Sensitivity_P25 = S_P25,

      I25_minus20 = I25_minus,
      I25_plus20 = I25_plus,
      Sensitivity_I25 = S_I25
    )
  )
}


# ============================================================
# RANK PARAMETERS BY ABSOLUTE AUC SENSITIVITY
# ============================================================

sensitivity_results <- sensitivity_results %>%
```

```r
  mutate(
    AbsSensitivity_AUC_B =
      abs(
        Sensitivity_AUC_B
      )
  ) %>%

  arrange(
    desc(
      AbsSensitivity_AUC_B
    )
  )


sensitivity_results$Rank_AUC_B <-
  seq_len(
    nrow(sensitivity_results)
  )


# ============================================================
# AUC TABLE
# ============================================================

auc_table <- sensitivity_results %>%
```

```
  select(
    Rank_AUC_B,
    Parameter,
    Baseline,
    AUC_B_minus20,
    AUC_B_plus20,
    Sensitivity_AUC_B
  )


# ============================================================
# IMMUNE-RELATED PARAMETERS
# ============================================================

immune_parameters <- c(
  "alpha",
  "epsilon",
  "K_D",
  "K_I",
  "K_N"
)


immune_table <- sensitivity_results %>%

  filter(
    Parameter %in% immune_parameters
```

```
  ) %>%

  arrange(
    desc(
      abs(
        Sensitivity_AUC_B
      )
    )
  )


# ============================================================
# COMPLETE TABLE
# ============================================================

complete_table <- sensitivity_results %>%

  select(
    Rank_AUC_B,
    Parameter,
    Baseline,

    AUC_B_minus20,
    AUC_B_plus20,
    Sensitivity_AUC_B,

    B25_minus20,
```

```
    B25_plus20,
    Sensitivity_B25,

    P25_minus20,
    P25_plus20,
    Sensitivity_P25,

    I25_minus20,
    I25_plus20,
    Sensitivity_I25
  )


# ============================================================
# VALIDATION OF SIMULATION TIME
# ============================================================

validation_results <- data.frame(

  Parameter = sensitivity_parameters,

  Minus20_final_time =
    NA_real_,

  Plus20_final_time =
    NA_real_
)
```

```
for (
  i in seq_along(
    sensitivity_parameters
  )
) {

  p <- sensitivity_parameters[i]


  parms_minus <- parms_base

  parms_minus[p] <-
    parms_base[p] * 0.80


  parms_plus <- parms_base

  parms_plus[p] <-
    parms_base[p] * 1.20


  sim_minus <- run_model(
    parms_minus
  )
```

```
  sim_plus <- run_model(
    parms_plus
  )


  validation_results$Minus20_final_time[i] <-
    max(
      sim_minus$time
    )


  validation_results$Plus20_final_time[i] <-
    max(
      sim_plus$time
    )
}


if (
  any(
    validation_results$Minus20_final_time < 25
  ) ||
  any(
    validation_results$Plus20_final_time < 25
  )
) {
```

```r
  stop(
    "At least one sensitivity simulation did not reach 25 h."
  )
}


# ============================================================
# PARAMETER LABELS FOR FIGURES
# ============================================================

parameter_labels <- c(

  r       = "italic(r)",
  K_C     = "italic(K)[C]",
  beta    = "beta",
  phi0    = "phi[0]",
  phi1    = "phi[1]",
  omega   = "omega",
  alpha   = "alpha",
  epsilon = "epsilon",
  K_D     = "italic(K)[D]",
  K_I     = "italic(K)[I]",
  K_N     = "italic(K)[N]",
  k_syn   = "italic(k)[plain(syn)]",
  K_A     = "italic(K)[A]",
  K_P     = "italic(K)[P]",
  H       = "italic(H)",
```

```
  tc      = "italic(t)[c]",
  k       = "italic(k)"
)


# =================================================================
# FUNCTION FOR UNICODE MINUS SIGN
# =================================================================

minus_sign_labels <- function(x) {

  labels <- format(
    x,
    trim = TRUE,
    scientific = FALSE
  )

  labels <- sub(
    "^-",
    "\u2212",
    labels
  )

  labels
}
```

```
# ===========================================================
# TORNADO PLOT DATA
# ===========================================================

tornado_data <- sensitivity_results %>%

  select(
    Parameter,
    Sensitivity_AUC_B
  ) %>%

  mutate(
    Parameter = reorder(
      Parameter,
      Sensitivity_AUC_B
    )
  )


# ===========================================================
# TORNADO PLOT
# ===========================================================

tornado_plot <- ggplot(

  tornado_data,
```

```
  aes(
    x = Parameter,
    y = Sensitivity_AUC_B
  )

) +

  geom_col() +

  coord_flip() +

  geom_hline(
    yintercept = 0,
    linetype = "dashed"
  ) +

  scale_y_continuous(
    labels = minus_sign_labels
  ) +

  scale_x_discrete(
    labels = function(x) {

      parse(
        text = parameter_labels[x]
      )
    }
```

```
) +

labs(
  x = "Parameter",
  y = "Local sensitivity coefficient"
) +

theme_minimal() +

theme(

  # Parameter labels
  axis.text.y =
    element_text(
      size = 16
    ),

  # Numerical values
  axis.text.x =
    element_text(
      size = 13
    ),

  # Axis titles
  axis.title.x =
    element_text(
      size = 15
```

```
    ),

    axis.title.y =
      element_text(
        size = 15
      ),

    # Remove minor grid lines
    panel.grid.minor =
      element_blank()
  )


print(
  tornado_plot
)


# ============================================================
# IMMUNE PARAMETER PLOT DATA
# ============================================================

immune_plot_data <- sensitivity_results %>%

  filter(
    Parameter %in% immune_parameters
  ) %>%
```

```
  mutate(
    Parameter = reorder(
      Parameter,
      Sensitivity_AUC_B
    )
  )


# ============================================================
# IMMUNE PARAMETER PLOT
# ============================================================

immune_plot <- ggplot(

  immune_plot_data,

  aes(
    x = Parameter,
    y = Sensitivity_AUC_B
  )

) +

  geom_col() +

  coord_flip() +
```

```
geom_hline(
  yintercept = 0,
  linetype = "dashed"
) +

scale_y_continuous(
  labels = minus_sign_labels
) +

scale_x_discrete(
  labels = function(x) {

    parse(
      text = parameter_labels[x]
    )
  }
) +

labs(
  x = "Immune-related parameter",
  y = "Local sensitivity coefficient"
) +

theme_minimal() +

theme(
```

```
# Parameter labels
axis.text.y =
  element_text(
    size = 16
  ),

# Numerical values
axis.text.x =
  element_text(
    size = 13
  ),

# Axis titles
axis.title.x =
  element_text(
    size = 15
  ),

axis.title.y =
  element_text(
    size = 15
  ),

# Remove minor grid lines
panel.grid.minor =
  element_blank()
```

```
  )


print(
  immune_plot
)


# ==============================================================
# EXPORT TABLES
# ==============================================================

write.csv(
  auc_table,
  "bacterial_AUC_sensitivity.csv",
  row.names = FALSE
)


write.csv(
  immune_table,
  "immune_parameter_sensitivity.csv",
  row.names = FALSE
)


write.csv(
```

```
  complete_table,
  "complete_local_sensitivity_analysis.csv",
  row.names = FALSE
)


write.csv(
  validation_results,
  "sensitivity_simulation_validation.csv",
  row.names = FALSE
)


# ============================================================
# EXPORT FIGURES
# ============================================================

ggsave(
  "bacterial_AUC_sensitivity_tornado.png",
  tornado_plot,
  width = 8,
  height = 6,
  dpi = 300
)


ggsave(
```

```
  "immune_parameter_sensitivity.png",
  immune_plot,
  width = 8,
  height = 5,
  dpi = 300
)


# ============================================================
# PRINT RESULTS
# ============================================================

print(
  auc_table,
  digits = 10,
  row.names = FALSE
)


print(
  immune_table,
  digits = 10,
  row.names = FALSE
)


cat(
```

```
  "\nParameters analyzed:",
  nrow(sensitivity_results),
  "\n"
)

cat(
  "Baseline bacterial AUC:",
  baseline_AUC_B,
  "\n"
)

cat(
  "Baseline B25:",
  baseline_B25,
  "\n"
)

cat(
  "Baseline P25:",
  baseline_P25,
  "\n"
)
```

```
cat(
  "Baseline I25:",
  baseline_I25,
  "\n"
)
```

Table S1. Complete local sensitivity analysis of Model 2.

Rank	Parameter	Baseline	AUCB (−20%)	AUCB (+20%)	Sensitivity coefficient
1	$φ_0$	6.00×10^{-8}	2,424,166.824	1,419,401.122	−1.320
2	r	1.00	1,563,159.993	2,053,553.213	+0.673
3	β	13.0	2,043,979.514	1,611,612.352	−0.586
4	ε	8.20×10^{-8}	1,933,839.835	1,703,771.615	−0.312
5	α	0.970	1,907,175.884	1,726,648.391	−0.245
6	tc	4.00	1,879,592.623	1,774,626.241	−0.142
7	KI	2.40×10^{7}	1,845,959.139	1,773,479.475	−0.099
8	ω	6.90×10^{-2}	1,775,140.604	1,820,291.018	+0.062
9	KD	2.20×10^{6}	1,818,154.632	1,782,355.849	−0.049
10	KN	1.00×10^{5}	1,784,528.979	1,811,517.998	+0.037
11	k	3.00	1,801,933.373	1,795,697.798	−0.009
12	KC	1.00×10^{8}	1,795,132.937	1,798,998.772	+0.005
13	$φ_1$	8.00×10^{-10}	1,797,823.315	1,797,082.650	−0.001
14	ksyn	42.5	1,797,690.090	1,797,220.252	−0.001
15	KA	5.81	1,797,357.611	1,797,530.766	+0.0002
16	H	1.00	1,797,541.553	1,797,376.443	−0.0002
17	KP	1.00×10^{6}	1,797,437.673	1,797,461.790	+0.00003
